# Post-breeding dispersal of nesting marine turtles from the NEOM Islands, Saudi Arabia

**DOI:** 10.1038/s41598-025-31237-1

**Published:** 2025-12-23

**Authors:** Hector Barrios-Garrido, Abdulrazaq Alatawi, Mishari Alghrair, Abdulaziz Alkaboor, Enjey Ghazzawi, Abdulqader Khamis, Brett Lyons, Paul Marshall, Abhishekh Palaparambil Vijaya, August Santillan, Deni Porej, Winston Cowie, Ricardo O. Ramalho

**Affiliations:** 1 KAUST Beacon Development, KAUST National Transformation Institute, Innovation Cluster, King Abdullah University of Science and Technology, 4700, 23955 Thuwal, Makkah Kingdom of Saudi Arabia; 2 NEOM Nature Reserve, Sharma 49631, Tabuk, Kingdom of Saudi Arabia; 3 Center for Ecology and Conservation, University of Exeter, Penryn, United Kingdom; 4 TropWATER - Centre for Tropical Water and Aquatic Ecosystem Research, James Cook University, Queensland 4811 Townsville, Australia

**Keywords:** Migratory pathways, Critical habitats, Habitat use, Marine protected areas, Red Sea, Animal migration, Animal behaviour, Herpetology, Ecology, Environmental sciences

## Abstract

Marine turtles, such as green (*Chelonia mydas*) and hawksbill (*Eretmochelys imbricata*), face numerous threats across their habitats. While they nest in the Red Sea, limited knowledge exists about their inter-nesting habitats and post-breeding movements. The NEOM Islands support ~ 95% of all documented nesting in the northeastern Red Sea, are crucial for turtle conservation. This study tracked 17 turtles (11 hawksbills, six greens) using platform terminal transmitters during two nesting seasons on Shusha and Walah Islands. We identified inter-nesting areas, migratory pathways, and foraging grounds, with displacements to foraging areas ranging from 34.8 to 501.7 km. Six feeding grounds were identified, four within Saudi Arabia and two in Egyptian waters. Hawksbills used broader inter-nesting habitats, particularly reef systems, emphasizing the need for conservation strategies targeting both nesting beaches and adjacent reefs. Our findings highlight the shared migratory routes of NEOM turtles with other Red Sea rookeries and support the importance of NEOM’s feeding and migration habitats for long-term conservation. The establishment of NEOM as a nature reserve will further enhance protection efforts for these species.

**Supplementary Information:**

The online version contains supplementary material available at 10.1038/s41598-025-31237-1.

## Introduction

Marine turtles are umbrella species that can be used to identify key environments used by other threatened taxa^1^. Their migratory pathways and feeding grounds are considered critical habitats that need to be protected^2^, with photic coral reefs, seagrass beds and sandy shorelines identified as critical habitats under the International Finance Corporation Performance Standard 6. Mapping marine turtle distributions is a key conservation action that can inform decision-takers to guide efforts to minimise human impacts and protect critical habitats^3^.

Marine turtles, including green (*Chelonia mydas*) and hawksbill (*Eretmochelys imbricata*) turtles, exhibit significant mobility and face various threats within their habitats^4–6^. Despite their nesting activities in the Red Sea, there is limited knowledge regarding their behaviours in inter-nesting habitats and post-breeding displacements from the Red Sea^7–11^. Recognising the importance of preserving marine turtle rookeries is crucial for attaining the objectives of the Kunming-Montreal Global Biodiversity Framework to stop and reverse biodiversity loss and living in harmony with nature, especially with threatened species.

This latter aspect is particularly significant within the NEOM Islands, which support ~ 95% of documented turtle nesting activity in the northeastern Red Sea, whereas nesting on the mainland remains limited^12^. NEOM is a visionary urban development project located along Saudi Arabia’s northwest Red Sea coast, encompassing an area of approximately 26,500 km^2^. As a cornerstone of Saudi Arabia’s Vision 2030 initiative, NEOM aims to diversify the nation’s economy by fostering innovation, sustainability, and technological advancement^13^.

Hence, exploring and understanding the dynamics of turtle movements in these key areas is essential for effective conservation strategies within Saudi Arabia and regionally in the Red Sea, and particularly within NEOM area. Therefore, utilising satellite tracking for post-nesting migrations serves as a robust initial step in understanding broader foraging distributions. Our study aims to identify the inter-nesting areas, migratory routes, and post-nesting migratory pathways and foraging geography of hawksbill and green turtles in NEOM, Saudi Arabia, from 2022 to 2023.

## Results

Over the course of the project, which encompasses two nesting seasons, 2022 for green turtles and 2023 for hawksbill turtles, a total of 17 turtles were assessed, tagged and monitored. This included six green turtles and eleven hawksbill turtles. All tagged turtles in this study were nesting females.

### Inter-nesting areas

**Fig. 1:**
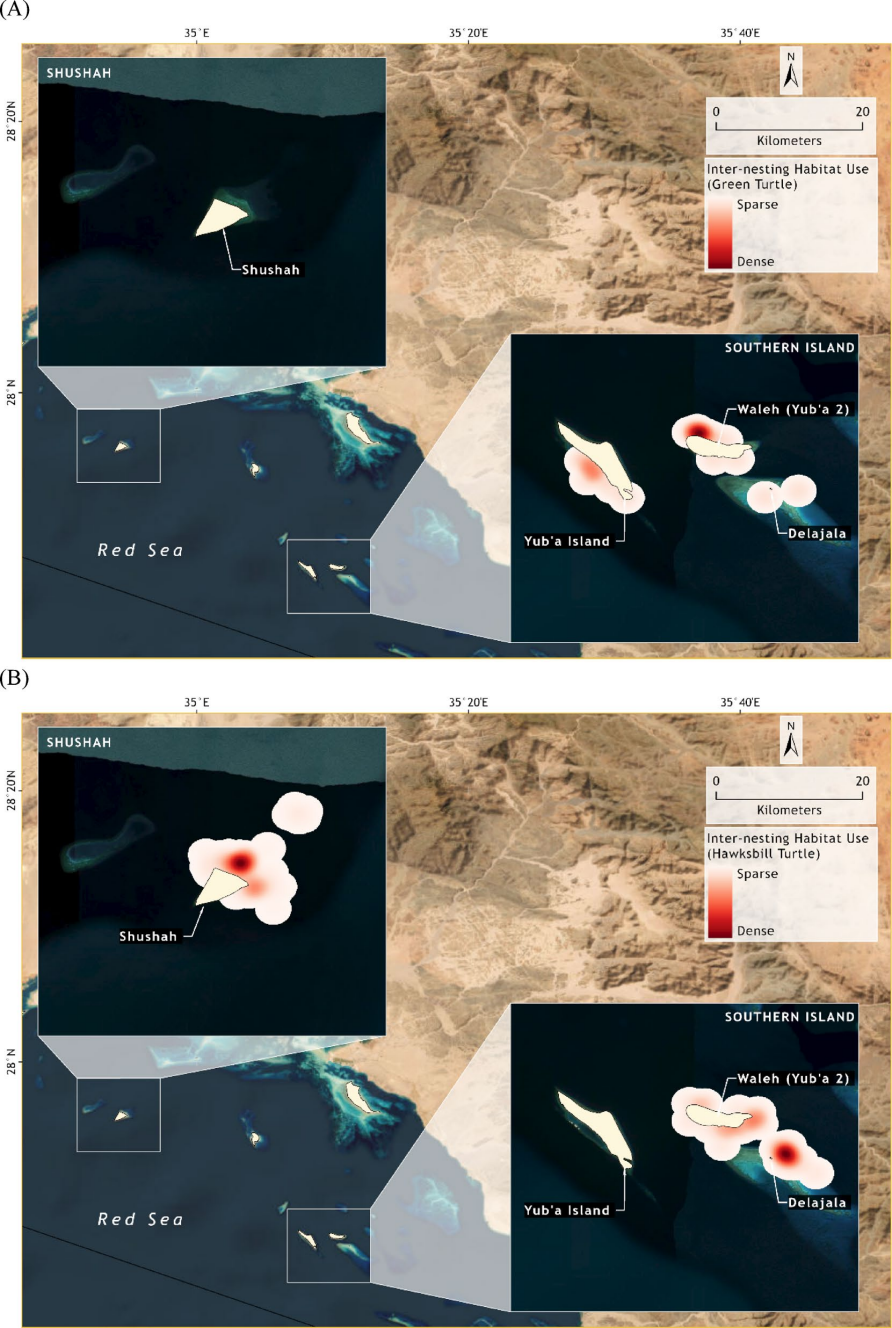
Inter-nesting habitat use of marine turtles (A = three green turtles; B = six hawksbill turtles) tagged at NEOM islands, Saudi Arabia.

We identified critical inter-nesting areas for both green turtles (*Chelonia mydas*) and hawksbill turtles (*Eretmochelys imbricata*) in the NEOM Islands during the 2022 and 2023 nesting seasons. Figure 1 presents the spatial distribution and inter-nesting habitat maps for green (Fig. 1a) and hawksbill (Fig. 1b) turtles, with all utilization distribution (UD) analyses conducted independently by species (See Supplementary Materials). Density of habitat use is shown from sparse (light) to dense (dark red). Inter-nesting UDs were calculated and analysed separately by species, and no pooled quantitative analysis was conducted.

The tagged green turtles frequently staying habitats close to their nesting beaches, primarily within a few kilometres of the shoreline. This preference was especially apparent at Walah Island (Fig. 1a) but also occurred nearby Yabu Island (in its post-reef lagoon, a shallow semi-enclosed lagoon located between the reef crest and the western coast of the island). These inter-nesting areas are essential for resting and preparing for subsequent nesting attempts. This limited range indicates that conservation efforts can be highly focused on these specific zones to ensure the protection of green turtle populations during their critical nesting period (e.g., habitat protection, go-slow and no-go zones).

Hawksbill turtles were primarily found and tagged at Shushah Island (seven), with some also found on Walah (three) and Delajala Islands (one) (Fig. 1b). Hawksbill turtles presented a wider range of inter-nesting habitats than did green turtles, covering broader areas, including reef systems around the islands. This indicates the need for a more extensive conservation strategy encompassing both nesting beaches and nearby reef habitats to effectively protect these turtles during their nesting and inter-nesting periods. In-water preliminary inspections confirmed that hawksbill turtles used reef system for both resting and opportunistic foraging (non-reproductive individuals), consistent with their behaviour at similar rookeries.

Despite the relatively small sample size, we successfully calculated the habitat UDs for hawksbill turtles in NEOM waters. The core areas (UD = 50%) observed for these turtles ranged from 0.73 km² to 2.71 km². The home ranges (UD = 95%) varied from 5.11 km² to 18.4 km² (Table 1) (Fig. S1, Fig. S2, Fig. S3). These findings provide valuable insights into the spatial ecology of hawksbill turtles in this region, highlighting the critical habitats utilised for their daily activities and emphasising the importance of these areas for conservation efforts. No UDs were calculated for the green turtles because of the restricted number of observations (See details in supplementary material – Table S1).

**Table 1:** Inter-Nesting habitat use (UD) core areas and home ranges for Hawksbill turtles satellite tagged at NEOM.

Species	Turtle ID (Flipper Tags)	PTT Code	CCL (cm)	Tagging Day (dd/mm/yyyy)	First Migration Day (dd/mm/yyyy)	Inter-nesting period studied (days)	Habitat Use (UDs)	
							Core Area (km^2^)	Home Range (km^2^)
*Eretmochelys imbricata*	RS-0475 RS-0476	0223945	74.6	11/06/2023	23/06/2023	12	2.55	18.4
	RS-0481 RS-0482	0223950	84.1	16/06/2023	02/07/2023	16	0.73	5.11
	RS-0489 RS-0490	0223954	71.2	19/06/2023	23/06/2023	4*	2.71	15.2

* Full dataset assessed as it was downloaded directly from the tag after the turtle carcase with the PTT was found

### Post-breeding dispersal and identified feeding grounds

**Fig. 2:**
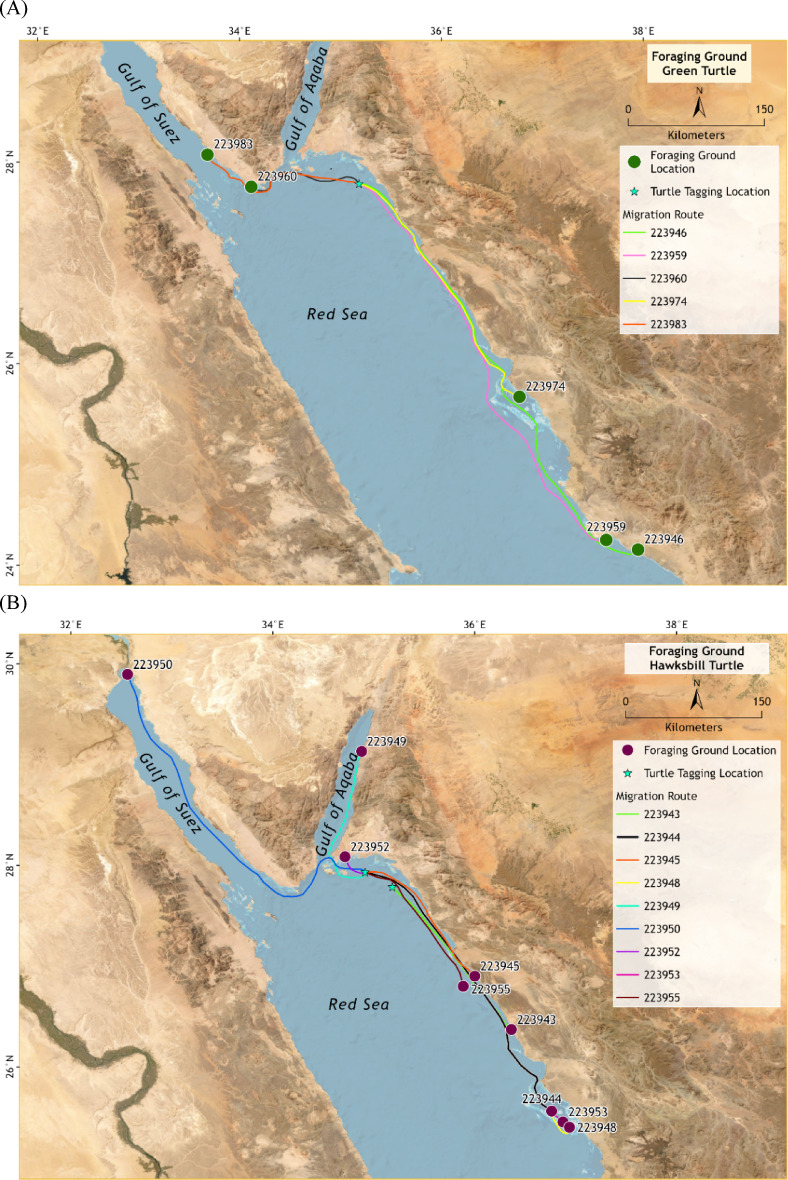
Post-nesting migration routes and foraging ground locations of marine turtles (A = five green turtles; and B = nine hawksbill turtles) tagged at NEOM Islands, Saudi Arabia. Tracks represent filtered FastGPS/Argos positions, with interpolated lines shown for clarity where transmission density was low. Foraging grounds are represented by utilisation distributions (UD 50% core and UD 95% home range).

The tracked migrations of these turtles resulted in displacements to foraging areas (Fig. 2). Green turtles migrated from the NEOM Islands to foraging grounds located between 34.8 km and 501.7 km from their nesting sites, with three individuals reaching feeding areas in Egyptian waters and the remaining individuals remaining within Saudi Arabian waters (Fig. 2a). On the other hand, Hawksbill turtles exhibited migration distances ranging from 135.6 km to 241.9 km, with most individuals establishing foraging grounds along the Saudi Arabian coastline (Fig. 2b). No individuals from either species dispersed beyond the Red Sea basin. These findings are crucial because they highlight the extensive migratory routes and the shared foraging grounds among turtles from the NEOM Islands and other rookeries in the southern Red Sea (Fig. S4, Fig. S5, Fig. S6, Fig. S7, Fig. S8, Fig. S9).

The core areas (UD = 50%) for the green turtles tagged at NEOM islands varied significantly, indicating individual variability in habitat usage. The core area values ranged from 0.23 km² to 1.77 km², with home range areas (UD = 95%) between 1.41 km² and 6.19 km² (Table 2) (Fig. S10, Fig. S11, Fig. S12). These findings reflect individual variation in spatial use during the foraging phase.

Hawksbill turtles demonstrated varied home range areas (UD = 95%) from 10.68 km² to 67.92 km² (median = 18.88 km²). The core areas (UD = 50%) for these turtles ranged from 1.91 km² to 15.05 km² (median = 3.22 km²) (Table 2) (Fig. S13, Fig. S14, Fig. S15, Fig. S16, Fig. S17, Fig. S18). Four of the six tagged turtles used well-defined areas, whereas two individuals showed a preference for broader habitats, possibly due to environmental factors or prey availability. Compared with hawksbill turtles tagged at the Red Sea Project, which had home ranges from 8.67 km² to 51.8 km² (mean = 27.76 km²), the ranges of the NEOM turtles included in this study were similarly extensive^14^.

**Table 2:** Foraging habitat use (UD) core areas and home ranges for green and Hawksbill turtles satellite tagged at NEOM.

Species	Turtle ID	PTT ID	Curved carapace length	Tagging day	Track days	Habitat use	
						Core areas (km^2^)	Home range (km^2^)
*Chelonia mydas*	RS-0458 RS-0461	0223946	106.6	06/09/2022	95	1.77	6.19
	RS_0456 RS-0457	0223959	96.7	06/09/2022	440	0.23	1.41
	RS-0471 RS-0472	0223983	102.1	22/09/2022	314	0.95	3.62
*Eretmochelys imbricata*	RS-0475 RS-0476	0223945	74.6	11/06/2023	162	1.91	12.17
	RS-0479 RS-0480	0223949	73.3	16/06/2023	157	5.88	27.16
	RS-0481 RS-0482	0223950	84.1	16/06/2023	157	2.66	19.73
	RS-0485 RS-0486	0223952	68.7	18/06/2023	155	2.39	10.68
	RS-0487 RS-0488	0223953	69.5	18/06/2023	146	3.79	18.04
	RS-0491 RS-0492	0223955	78.1	20/06/2023	153	15.05	67.92

The green turtles presented distinct feeding grounds post-nesting (Fig. 2a). For example, turtle PTT-0223974 migrated from Walah Island to Alash Shargi Island (inside the Al-Walh Lagoon) (Fig. S5), and turtle PTT-0223960 moved from northern Walah Island to the Suez Channel near the Ras Mohamed Natural Reserve (Egypt) (Fig. S6). Another turtle, PTT-0223983, utilised feeding grounds inside the Gulf of Suez (Egypt) (Fig. S12). These migrations covered substantial distances, highlighting the importance of specific areas for their foraging activities (Fig. S10, Fig. S11, Fig. S12).

The identified feeding grounds for hawksbill turtles varied notably (Fig. 2b). Turtle PTT-0223945’s feeding grounds at Zubaida Island (close to Duba city, Saudi Arabian territory) and turtle PTT-0223955’s broader area usage highlight diverse habitat preferences. The distances between the nesting and feeding sites ranged from 135.61 km to 241.92 km, emphasising significant migratory behaviour for foraging. The home ranges for the feeding areas were also extensive, with median values reflecting a broad usage pattern across different habitats (Table 2) (Fig. S13, Fig. S14, Fig. S15, Fig. S16, Fig. S17, Fig. S18).

## Discussion

The results of this study underscore the significant mobility of green and hawksbill turtles from the NEOM Islands, as evidenced by their extensive migration to foraging grounds across the Red Sea. The identification of six primary foraging grounds (based on 12 final locations), including sites within both Saudi Arabian and Egyptian waters, suggests regional connectivity and highlights the critical need for transboundary and national conservation efforts. Three turtles tracked in this study migrated into the designated Prince Mohammed bin Salman Royal Reserve, and two others remained resident within NEOM waters, this highlights the importance of both areas for long-term turtle conservation. All the areas identified in this study offer a valuable basis to guide future conservation and marine spatial planning efforts in NEOM and the Red Sea region and its other giga-projects.

Expanding existing marine protected areas (MPAs) or creating new areas in the Red Sea coastal zone could greatly benefit the conservation of these species during and after their reproductive episodes^11,12^. The shared migratory routes and foraging areas indicate that conservation actions in one region could have positive repercussions across the broader migratory network of these turtles. MPAs can provide safe havens where turtles can feed, breed, and nest without the threats posed by human activities such as coastal development, fishing, and pollution. Effective MPAs should be strategically located to encompass critical habitats identified in this study, including nesting beaches, inter-nesting habitats, and foraging grounds^2^. The design of 95% NEOM as a nature reserve will significantly increase marine turtle conservation efforts for both studied species, benefiting them during and after their reproductive periods.

This study also emphasises the importance of continued research to fill knowledge gaps regarding the inter-nesting and post-breeding behaviours of marine turtles (Fig. 1). Long-term monitoring and tracking studies are crucial for understanding the full extent of their migratory patterns and habitat use^12^. Such data are invaluable for informing adaptive management strategies and ensuring that conservation efforts are based on the best available science^2,5,15^. The data presented here have informed immediate conservation actions and long-term management plans for the NEOM Islands, aimed at protecting and sustaining marine turtle populations in this critical habitat.

Migration routes highlight both nearshore residency and long-distance movements to distinct feeding grounds (Fig. 2). The shared migratory routes and foraging areas between turtles from the NEOM Islands and other rookeries in the region further highlight the interconnected nature of marine turtle populations^9,11,14,15^. Conservation actions in one region can have positive repercussions across the broader migratory network of these turtles. Thus, regional cooperation and coordinated management strategies are essential to address the conservation needs of these migratory species effectively.

By identifying these key inter-nesting areas, this study provides valuable insights for developing targeted conservation actions with a dynamic approach aimed at preserving the delicate balance of marine ecosystems in the NEOM region, ultimately contributing to the sustainability of these endangered species. Our findings highlight the need for coordinated transboundary conservation efforts, as turtles from NEOM utilize foraging grounds in both Saudi Arabian and Egyptian waters, demonstrating the interconnectedness of marine ecosystems across the Red Sea region.

In conclusion, the findings from this study provide valuable insights into the migratory strategies and foraging geography of green and hawksbill turtles in the NEOM Islands. These insights are essential for guiding effective conservation strategies and ensuring the protection of critical habitats necessary for the survival of these threatened species.

The data from this study will provide valuable guidance for further refinement of NEOM’s marine zoning plan, including protection and regulation of vessel speed and the timing and intensity of uses of critical habitats beyond nesting beaches. The findings also underscore the importance of developing a regional, international approach to sea turtle conservation and synchronising conservation efforts across the northern Red Sea region as soon as possible.

## Methods

**Fig. 3:**
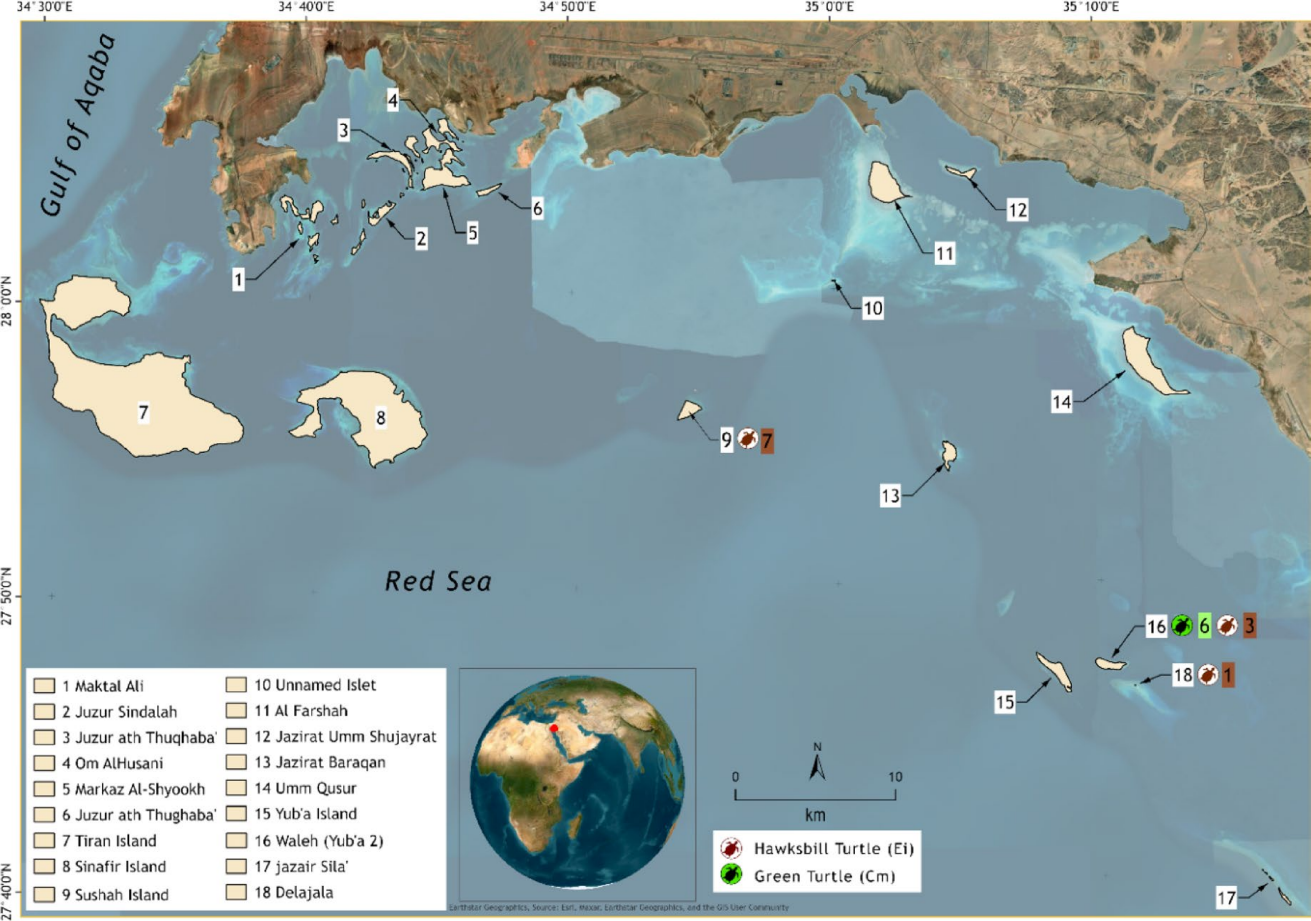
Study area and tagging locations of marine turtles at the NEOM Islands, northeastern Red Sea, Saudi Arabia.

Field surveys were carried out at the NEOM Islands in the Saudi Arabian Red Sea (Fig. 3). Located in the northeastern portion of the Red Sea, the NEOM Islands were surveyed as part of a comprehensive conservation and monitoring program managed by the NEOM Nature Reserve and led in-field by the Terrestrial Ecology staff of the Beacon Development Department of the King Abdullah University of Science and Technology (KAUST).

Forty-four surveys were carried out across two nesting seasons (2022 and 2023). The effort consisted of 32 night-time beach surveys focused on observing and tagging nesting turtles and 12 daytime surveys to detect nesting evidence and perform free-dive captures.

Nocturnal surveys were carried out primarily to observe and then tag nesting turtles once they had actively engaged in nesting behaviour. This included flipper tagging and deployment of platform terminal transmitter (PTT) devices. The tagging allowed for continuous tracking of these turtles throughout and beyond the breeding season. PTT tracking aims to track the movements of individual turtles over an extended period, providing valuable insights into their nesting behaviours and post-breeding season dispersal^2^ (See details in supplementary material – Table S1).

We deployed PTT tags, commonly referred to as satellite tags, to track 17 turtles in total. Six adult female green turtles were nesting (found and tagged at Walah Island from September-2022), and 11 adult female hawksbill turtles were nesting or resting (in-water) at Shushah, Walah, and Delajala Islands (one from September-2022 and 10 from June-2023; Table 3). Before the PTTs were set, the turtles were examined to corroborate their health status, and curved carapace length (CCL) measurements were taken. All PTTs were attached following the standardized protocol described in Mann et al.^15^, which involved cleaning the carapace, applying marine epoxy and fiberglass strips, and coating the device with antifouling paint as recommended by the PTT manufacturer.

**Table 3:** Summary of PTT deployments for 17 nesting turtles found within the NEOM territory.

PTT code	Tagging date (dd/mm/yyyy)	Island	Habitat captured	Days tracked	Raw fixes	Locations fixes			Curved CarapaceLenght (cm)
						Inter-nesting	Migration	Foraging	
*Chelonia mydas*									
0223946	06/09/2022	Walah	Nesting	95	55	6	1	46	106.6
0223947	09/09/2022	Walah	Nesting	31	10	6	0	0	98.2
0223959	06/09/2022	Walah	Nesting	440	426	2	11	411	96.7
0223960	13/09/2022	Walah	Nesting	187	28	5	2	17	104.9
0223974	12/09/2022	Walah	Nesting	84	26	7	11	2	110.4
0223983	22/09/2022	Walah	Nesting	314	123	2	1	116	102.1
*Eretmochelys imbricata*									
0223943	10/06/2023	Walah	Nesting	12	27	9	11	0	69.1
0223944	08/09/2022	Delajala	Nesting	43	15	8	3	2	57.4
0223945	11/06/2023	Walah	In-Water	162	319	27	15	261	74.6
0223948	11/06/2023	Walah	Nesting	31	28	0	16	11	71.4
0223949	16/06/2023	Shushah	Nesting	157	258	13	34	203	73.3
0223950	16/06/2023	Shushah	Nesting	157	171	25	59	79	84.1
0223951	17/06/2023	Shushah	Nesting	9	43	16	21	0	71.1
0223952	18/06/2023	Shushah	Nesting	155	264	9	11	241	68.7
0223953	18/06/2023	Shushah	Nesting	155	288	2	38	247	69.5
0223954	19/06/2023	Shushah	Nesting	4	67	65*	0	0	71.2
0223955	20/06/2023	Shushah	Nesting	153	221	8	102	105	78.1

*elevated number of locations as all the data were directly downloaded from the retrieved tag. Natural mortality registered (probably shark predation).

Satellite tags were attached and the full tagging procedure was completed within two hours of first encountering each turtle, after which the animal was released at the same location. The PTT device model used was the F6G-376B from Lotek (Havelock North, New Zealand) (https://www.lotek.com), which transmits both Argos and Fast GPS location information via the Argos satellite system. All transmitters were programmed by default with a two-phase duty cycle to optimize data acquisition during critical behavioural phases while conserving battery life for long-term tracking (See details in supplementary material – Table S2). This configuration provided high-resolution location data during inter-nesting and early migration periods, while allowing sufficient battery reserves to capture dispersal and foraging behaviour in longer deployments, should they occur.

The PTTs sent messages to a satellite each time the turtles came to the surface. The received data were relayed by a satellite that includes information on the location of the turtle. The data were plotted onto a map to identify movements that were monitored over an extended period^16^. The duration of data collection varied among individuals (Table 3). The data transmitted by the PTTs lasted between 4 and 440 days.

Certain recorded coordinates exhibit inaccuracies, potentially due to various factors, such as sea state, cloud cover, or satellite availability^15^. Consequently, the database was filtered before analysis. Biologically unrealistic locations, such as points in terrestrial areas, were eliminated via the R package SDL filter^17,18^. Additionally, duplicate locations were removed to maintain a single point per time and location, thereby avoiding pseudoreplication^2,3,19^. Through a visual assessment of the plotted tracks and the spatiotemporal changes in locations, general use areas were identified.

These areas were classified as ‘nesting’, ‘inter-nesting’, or ‘foraging’ areas, separated by nesting movements (between nesting and inter-nesting areas) or post-breeding migrations (between nesting or inter-nesting and foraging areas). To distinguish behavioural phases, we used a combination of visual inspection and movement tortuosity thresholds, based on the angle between sequential locations^15^. A shift to low tortuosity (angles > 150°) with consistent directional movement marked the onset of post-nesting migration. Foraging grounds were identified when turtles exhibited high tortuosity (angles < 120°) and remained within a localized area for ≥ 15 days. This angle-based segmentation approach follows methods outlined by Mann et al.^15^ and enabled robust phase classification despite variable track durations. This approach allowed us to distinguish phases with improved confidence and reduced misclassification of transient locations as foraging grounds.

Location data were filtered to ensure spatial accuracy and biological realism. For Argos data, only high-quality location classes (LC 1, 2, 3) were used; lower classes (0, A, B, Z) were excluded. FastGPS fixes were limited to high-confidence positions (< 100 m error), based on internal quality diagnostics. A speed filter (5 km/h) was applied to remove improbable movements, and all tracks were visually checked before analysis. The filtered locations were expected to be within 47.1 ± 61.0 m (mean ± SD) of the true locations. These parameters, standardised by previous authors for hard-shell marine turtles, account for the time lag between subsequent locations to estimate the distribution surface by weighting the time period between locations^3,9,20–22^. Although this accuracy estimate is specific to Fastloc-GPS devices, and FastGPS has its own error structure [see details in 23] using Shimada et al.’s^10^ thresholds provides a practical and conservative filtering approach that is unlikely to overestimate utilisation distributions or materially change the interpretation of habitat use. This method is well suited for estimating home ranges, as it considers the uneven temporal sampling of locations due to transmitter characteristics and turtle diving behaviour during migration and feeding^6,15,20–22^.

The distance between the initial location on the nesting beach and the final location at the foraging site was calculated via Google Earth for each turtle. This calculation utilised the release location on the nesting beach and the last point transmitted and decoded in the Lotek data portal.

The SDL filter package in R was employed to refine the collected data, which were subjected to rigorous filtering to eliminate inaccuracies due to factors such as sea state, cloud cover, or satellite availability. The filtering process ensured that biologically unrealistic locations (e.g., terrestrial points) were excluded, and duplicate locations were removed to maintain one accurate point per time and location.

All monitoring activities were permitted under the King Abdullah University of Science and Technology (KAUST) Institutional Animal Care and Use Committee (IACUC) permit No. 18IACUC11 and 22IACUC05. Transmitters (Lotek FastGPS Argos, model F6G-376B) were selected based on species size and life stage, and their mass did not exceed 3% of the turtle’s body mass, in accordance with established ethical guidelines for satellite tagging of marine turtles.

## Supplementary Information

Below is the link to the electronic supplementary material.

Supplementary Material 1

Supplementary Material 2

Supplementary Material 3

## Data Availability

The data supporting this study’s findings are available from the corresponding author upon reasonable request and with permission from NEOM.
