# Supplementary material for: Post-breeding dispersal of nesting marine turtles from the NEOM Islands, Saudi Arabia
Source: Sci Rep. 2025 Dec 23;16:1695. doi: 10.1038/s41598-025-31237-1 (PMC12800070; doi:10.1038/s41598-025-31237-1)
Supplement: Supplementary file 1 — Supplementary Material 1 [file 41598_2025_31237_MOESM1_ESM.docx]

Supplementary Materials for the article:

**Dispersal of nesting marine turtles from the NEOM Islands, Saudi Arabia, after breeding**

Hector Barrios-Garrido^1^; Abdulrazaq Alatawi^1^; Mishari Alghrair^2^; Abdulaziz Alkaboor^1^; Enjey Ghazzawi^2^; Abdulqader Khamis^2^; Brett Lyons^2^; Paul Marshall^2^; Abhishekh Palaparambil Vijaya^1^; August Santillan^1^; Deni Porej^2^; Winston Cowie^2^; Ricardo O. Ramalho^1^

1 KAUST Beacon Development; KAUST Innovation; King Abdullah University of Science and Technology (KAUST). Thuwal, Makkah, 23955; Kingdom of Saudi Arabia.

2 NEOM. Sharma 49631; Kingdom of Saudi Arabia.

Corresponding author: [hector.barrios@kaust.edu.sa](mailto:hector.barrios@kaust.edu.sa)

This section contains maps of the foraging sites, including their home ranges (UD 95%) and core areas (UD 50%) from the turtles tagged in this study.

This file also contain maps illustrating movement patters of tagged turtles, highlighting the tagging and last transmitted locations. Transmission points are color-coded, with older locations in yellow and recent locations of the transmitted fixes in orange.

The black arrows represent the turtle movement direction (not necessarily the migratory route). It is represented on that way due to limitations in the data transmitted by the PTTs between tagging locations and final (potential feeding) location.

An additional table with the duty cycle configuration used is included here.


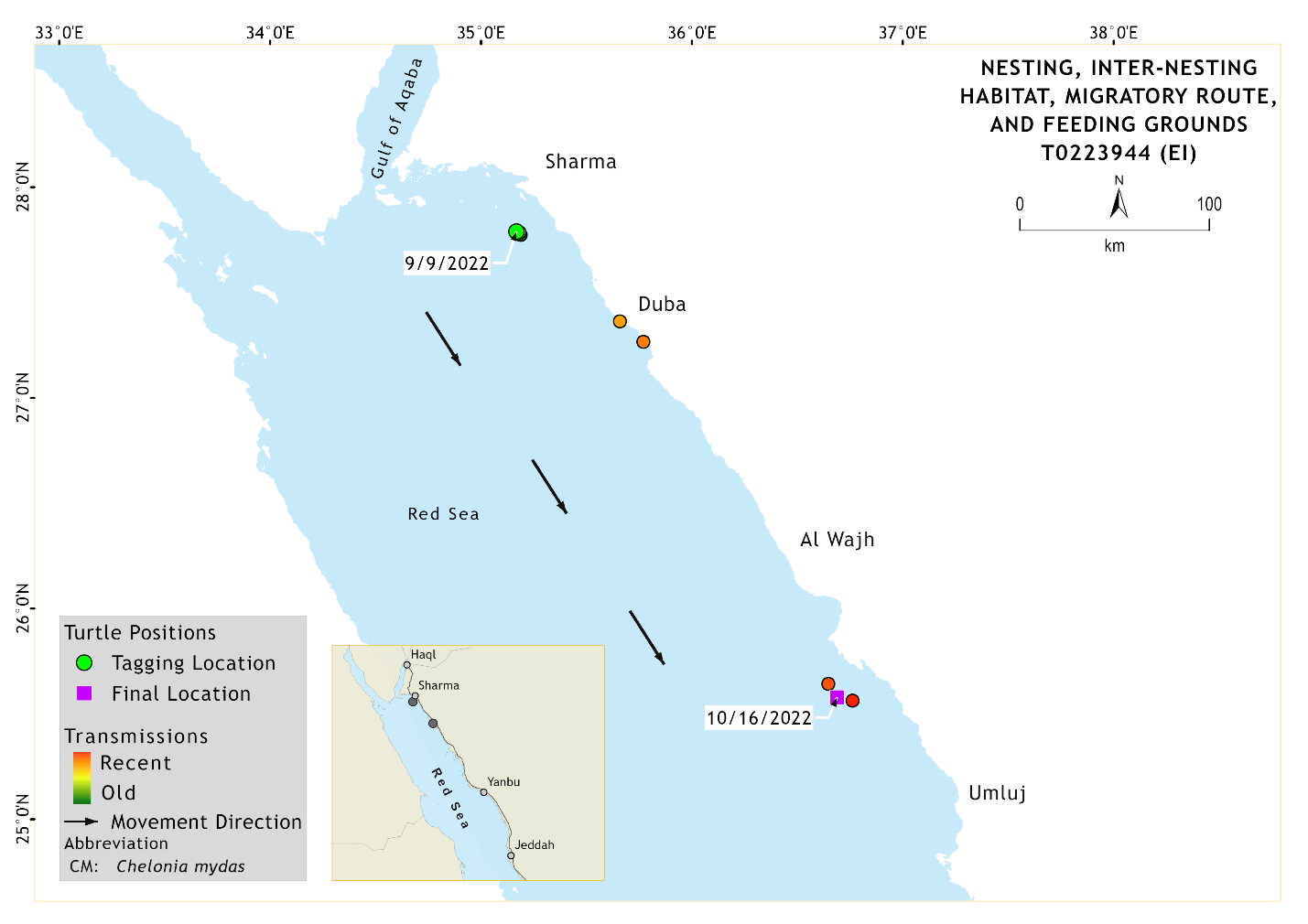

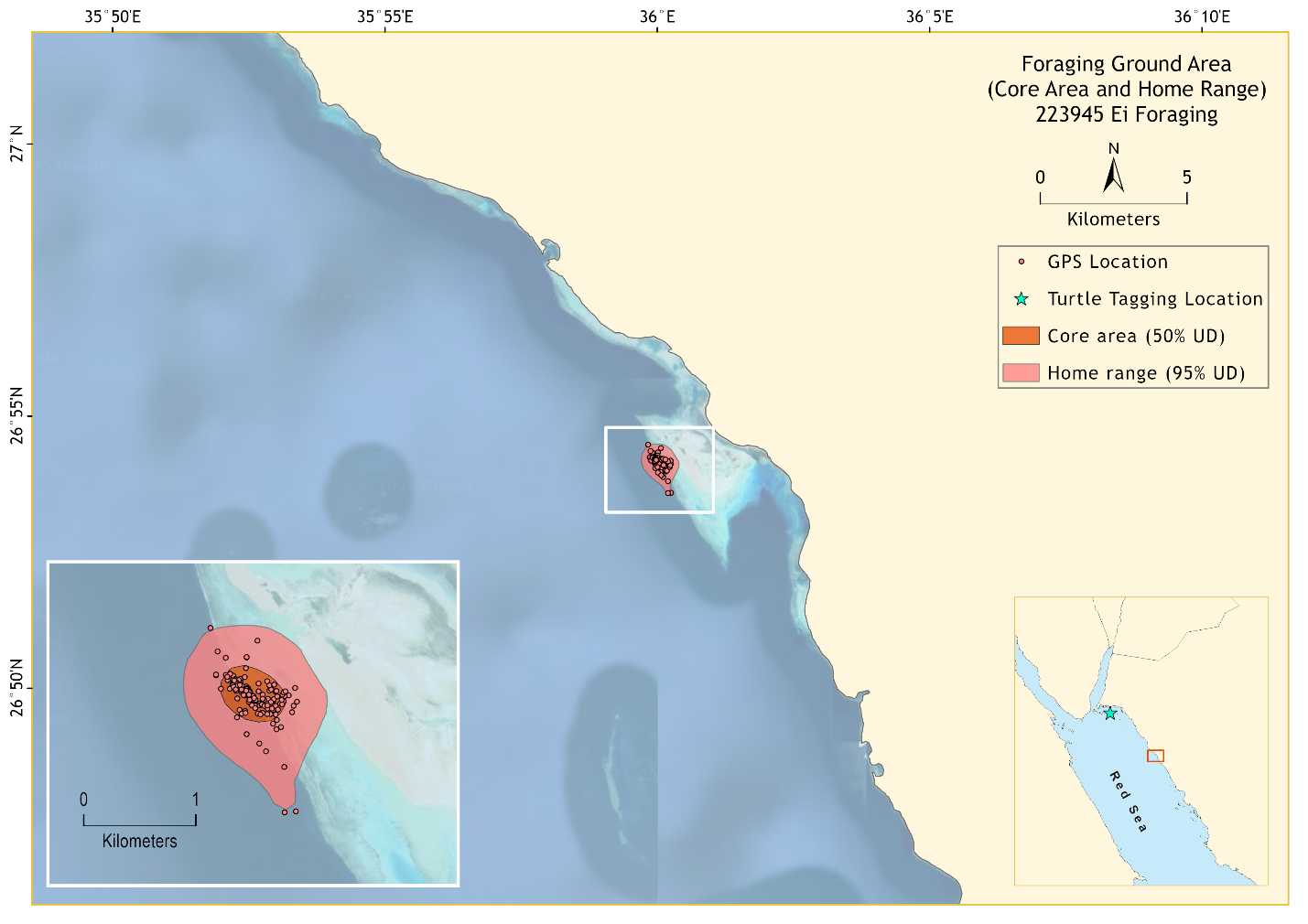

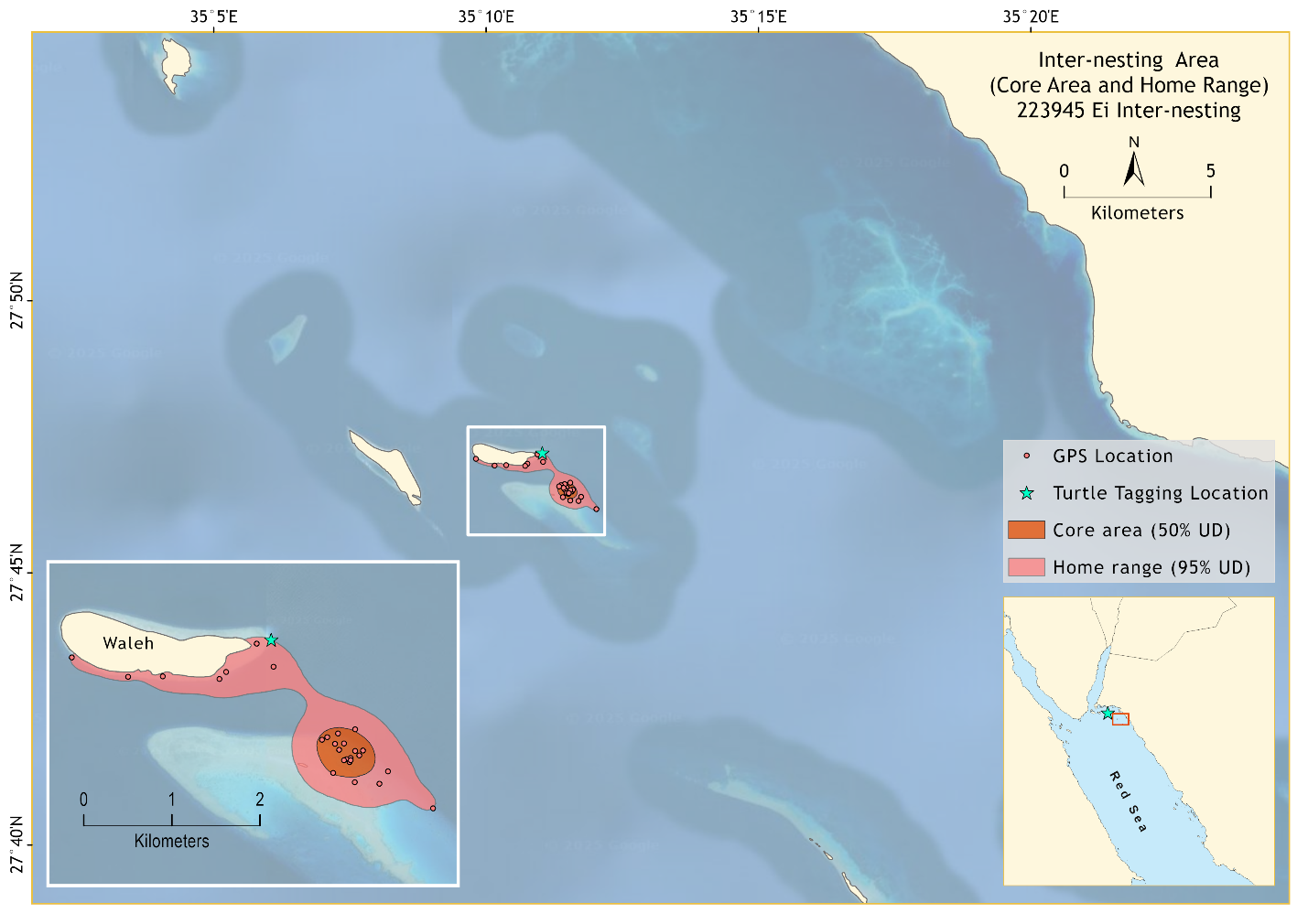

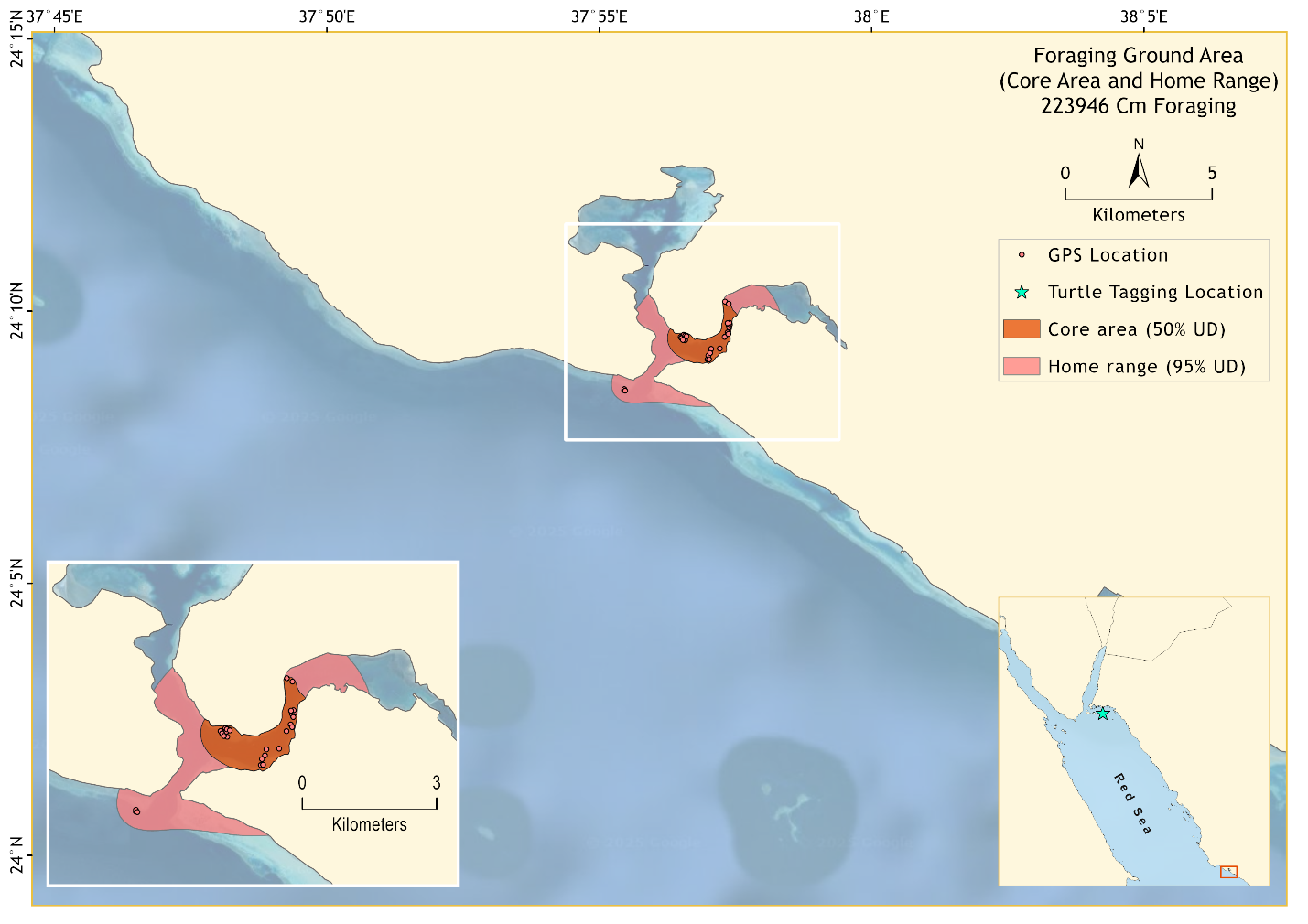

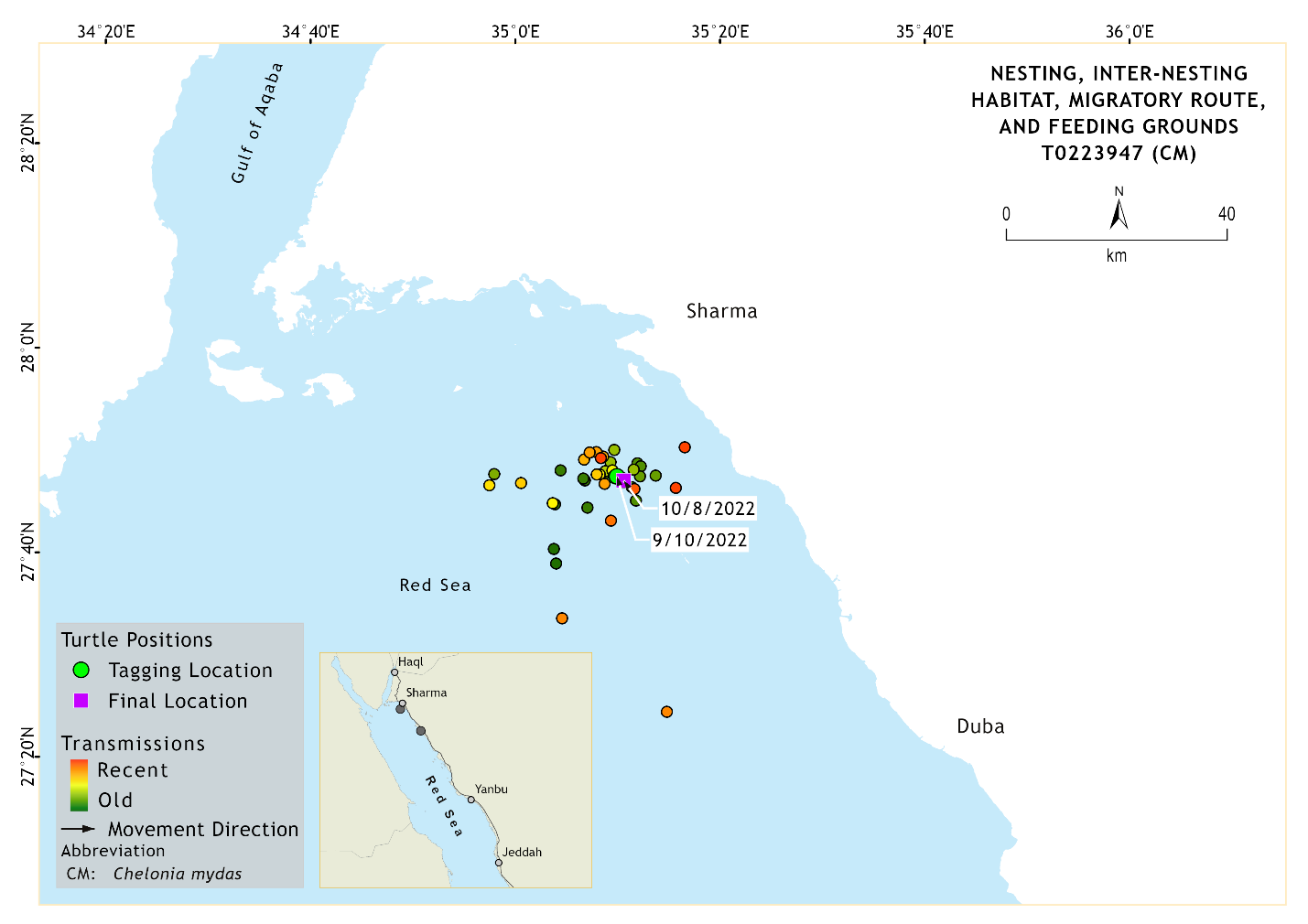

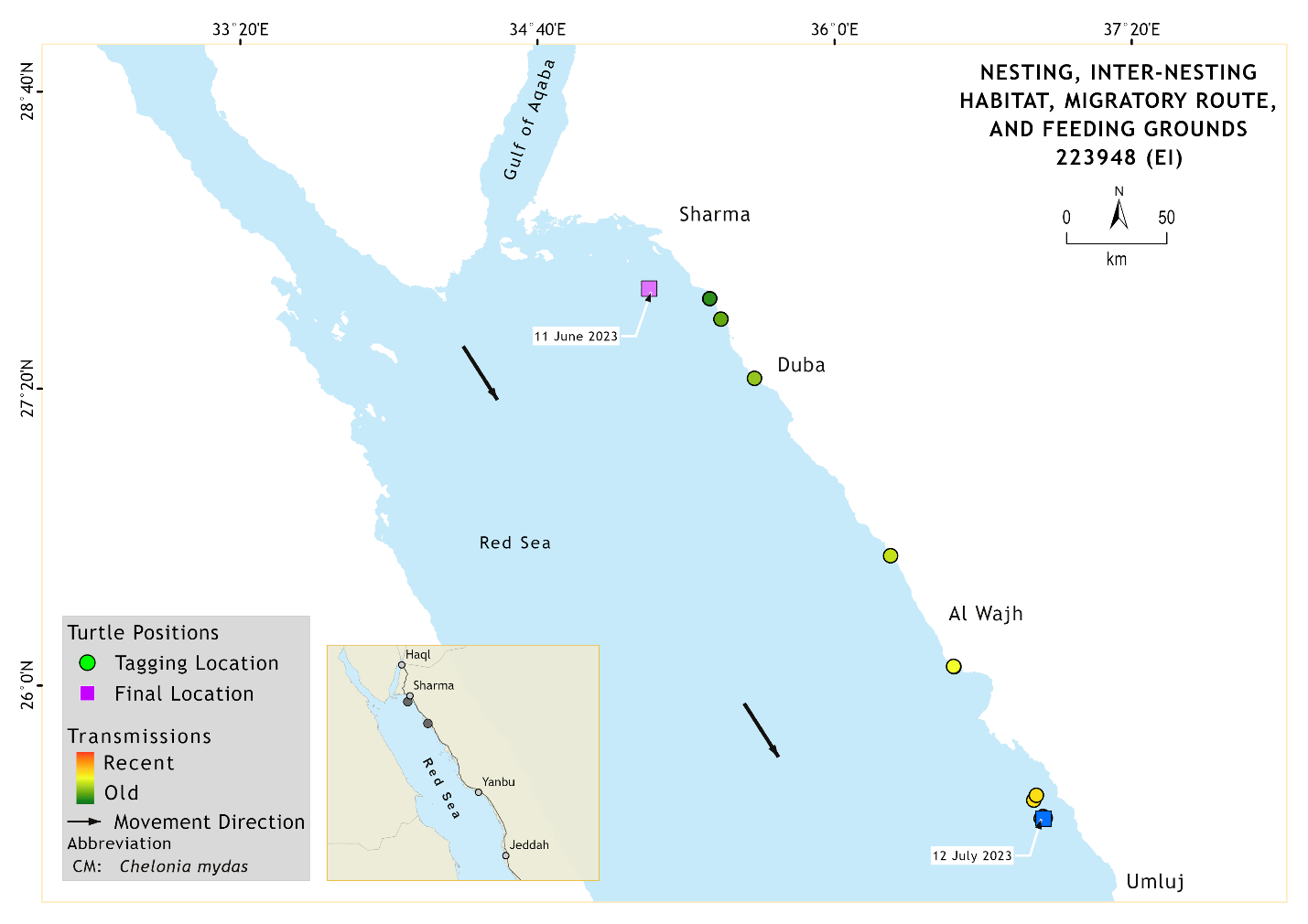

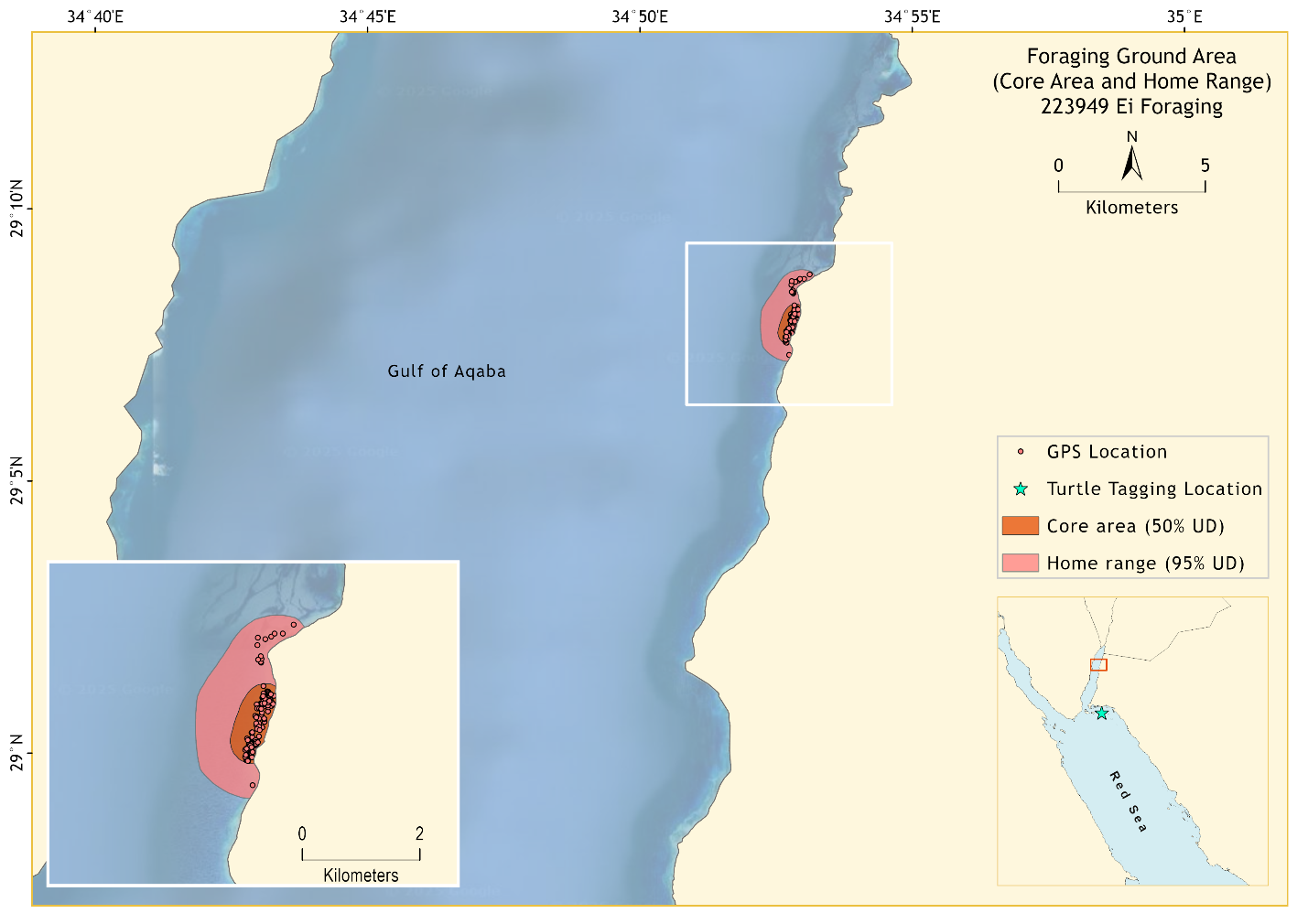

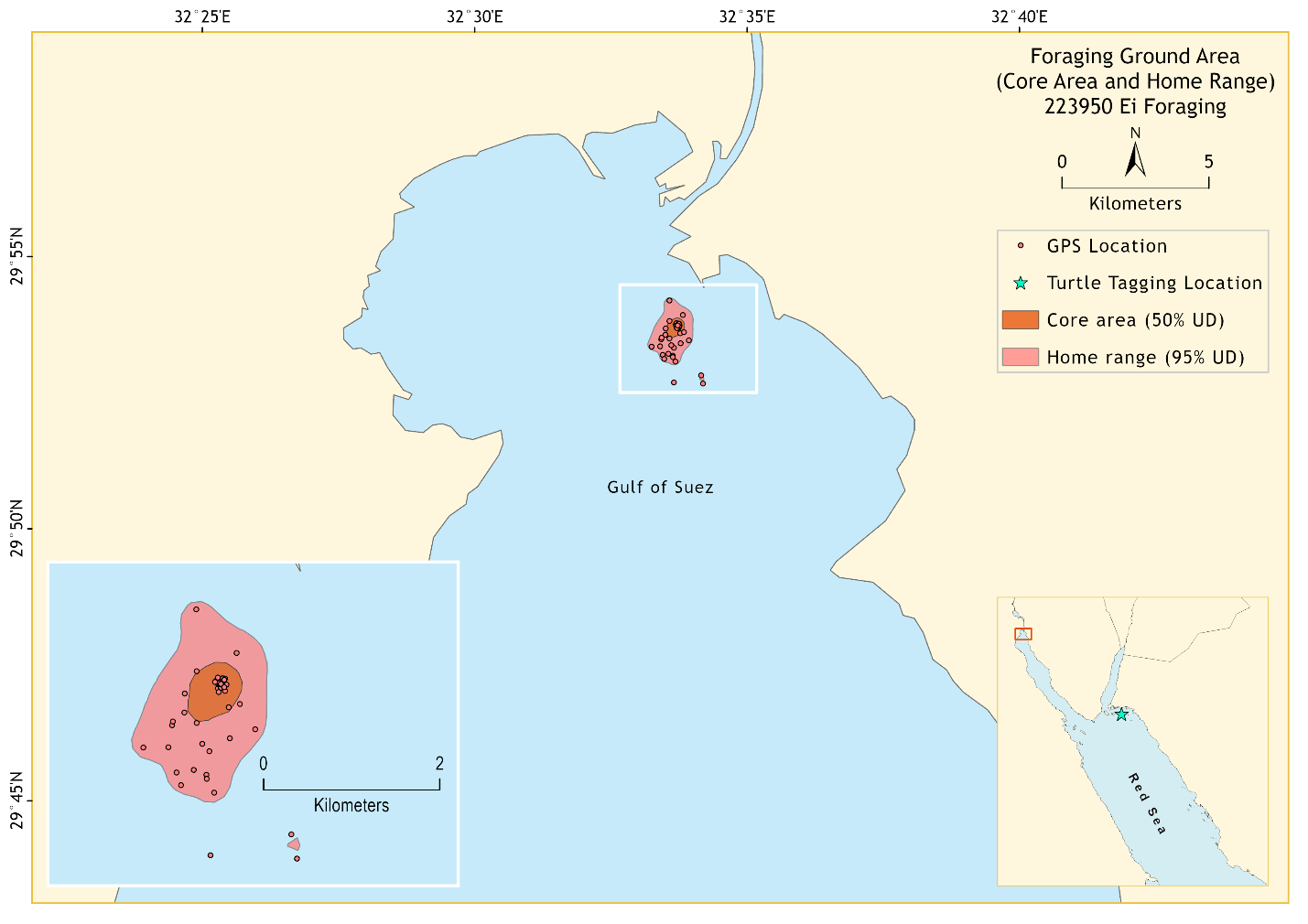

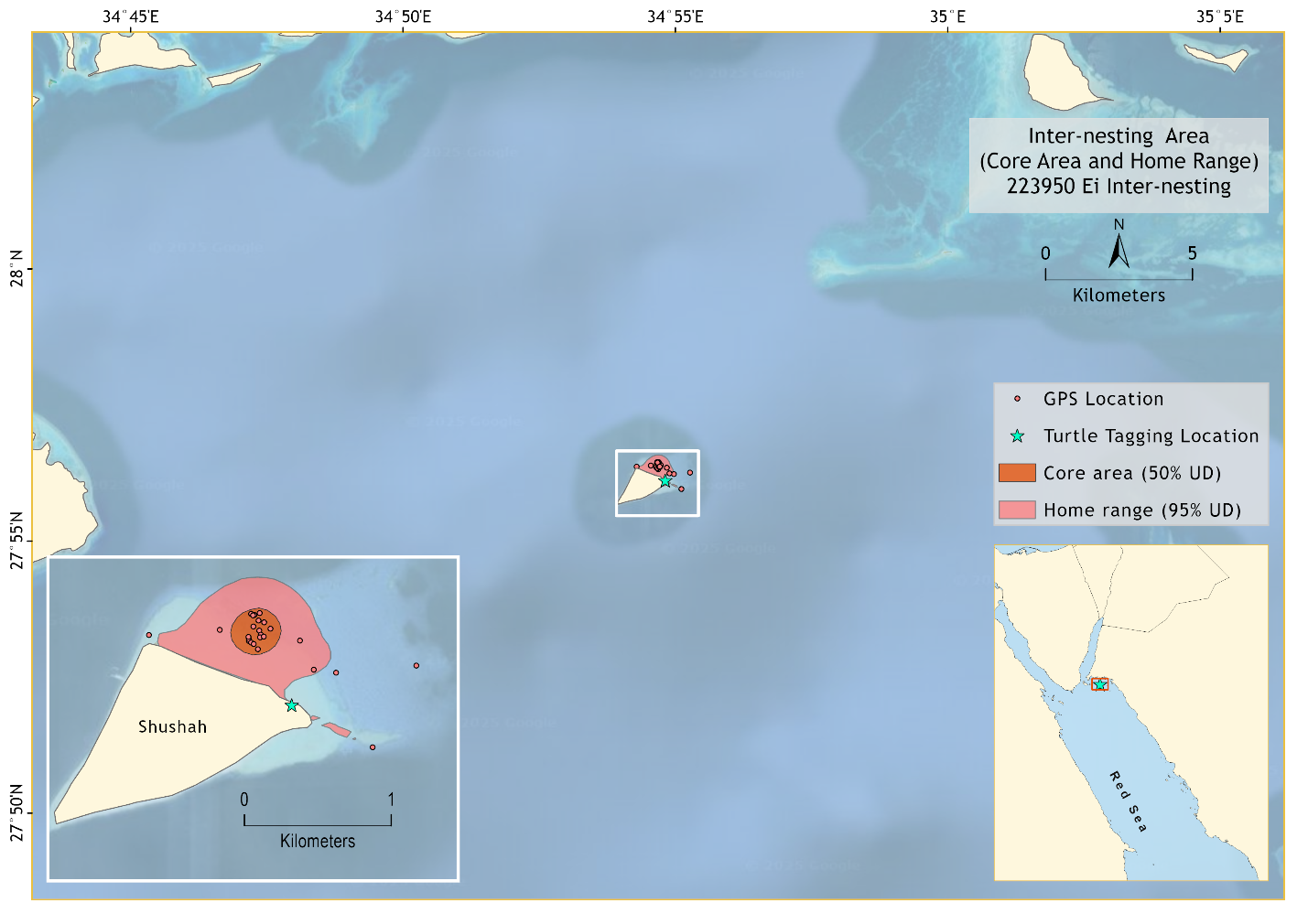

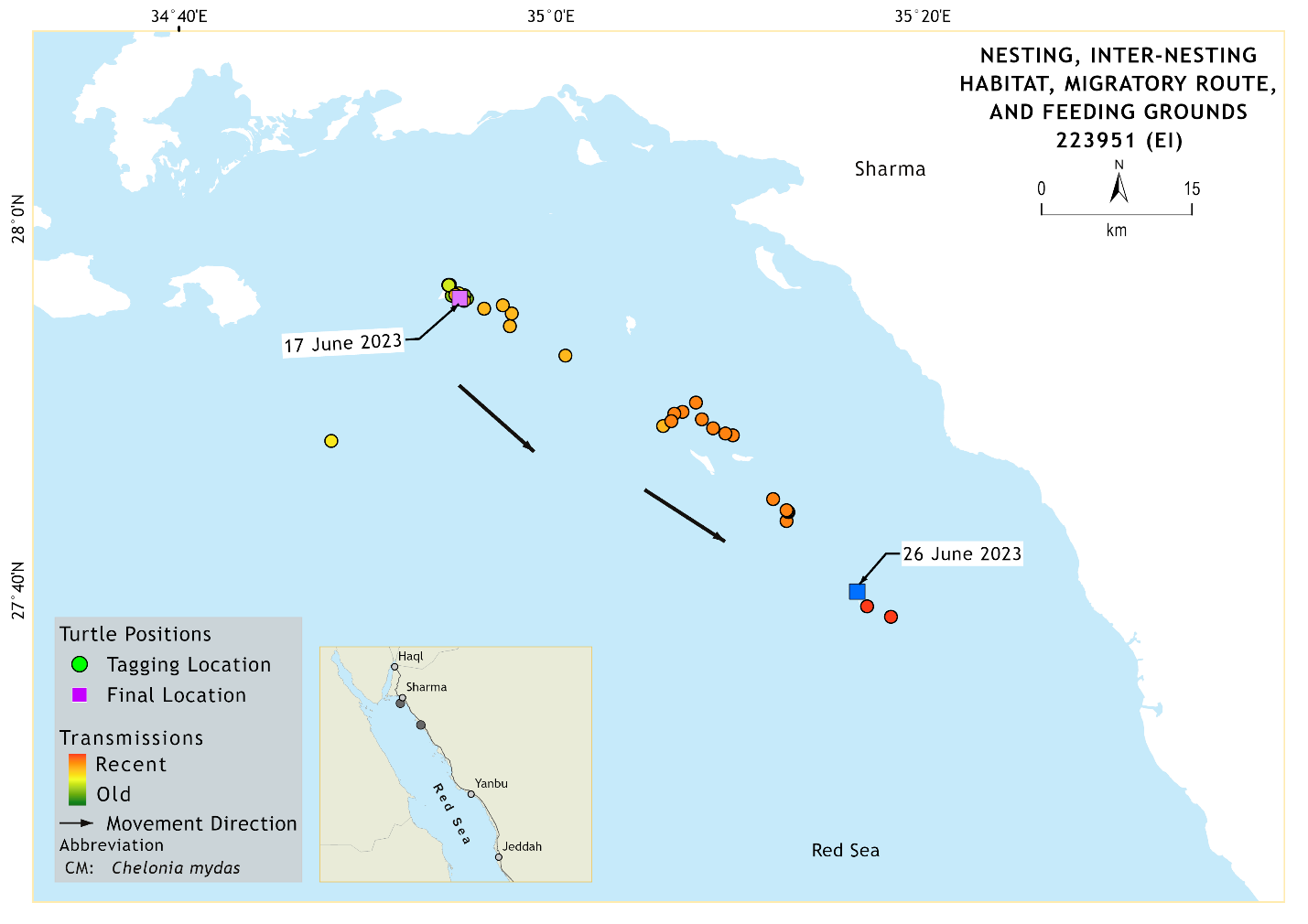

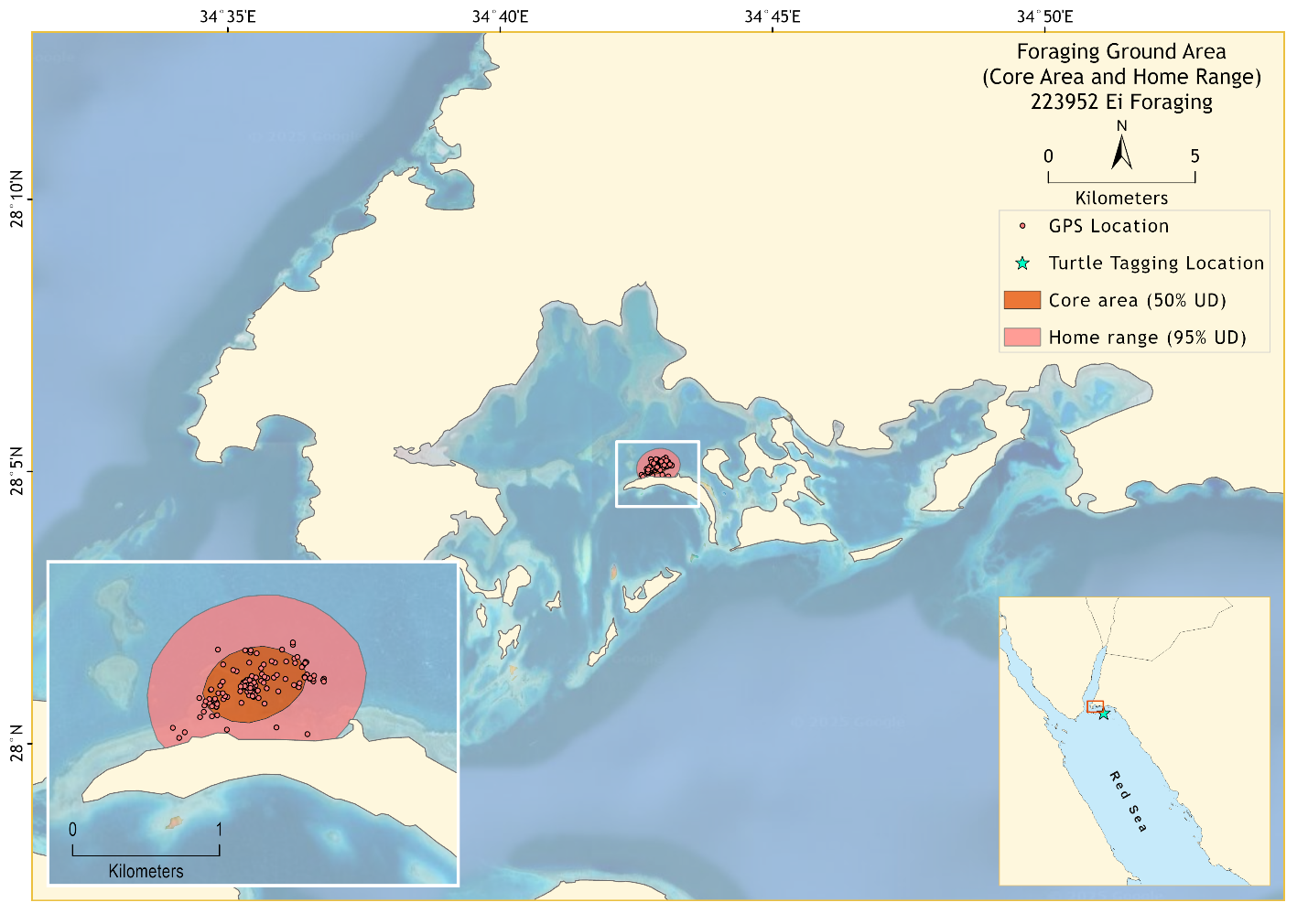

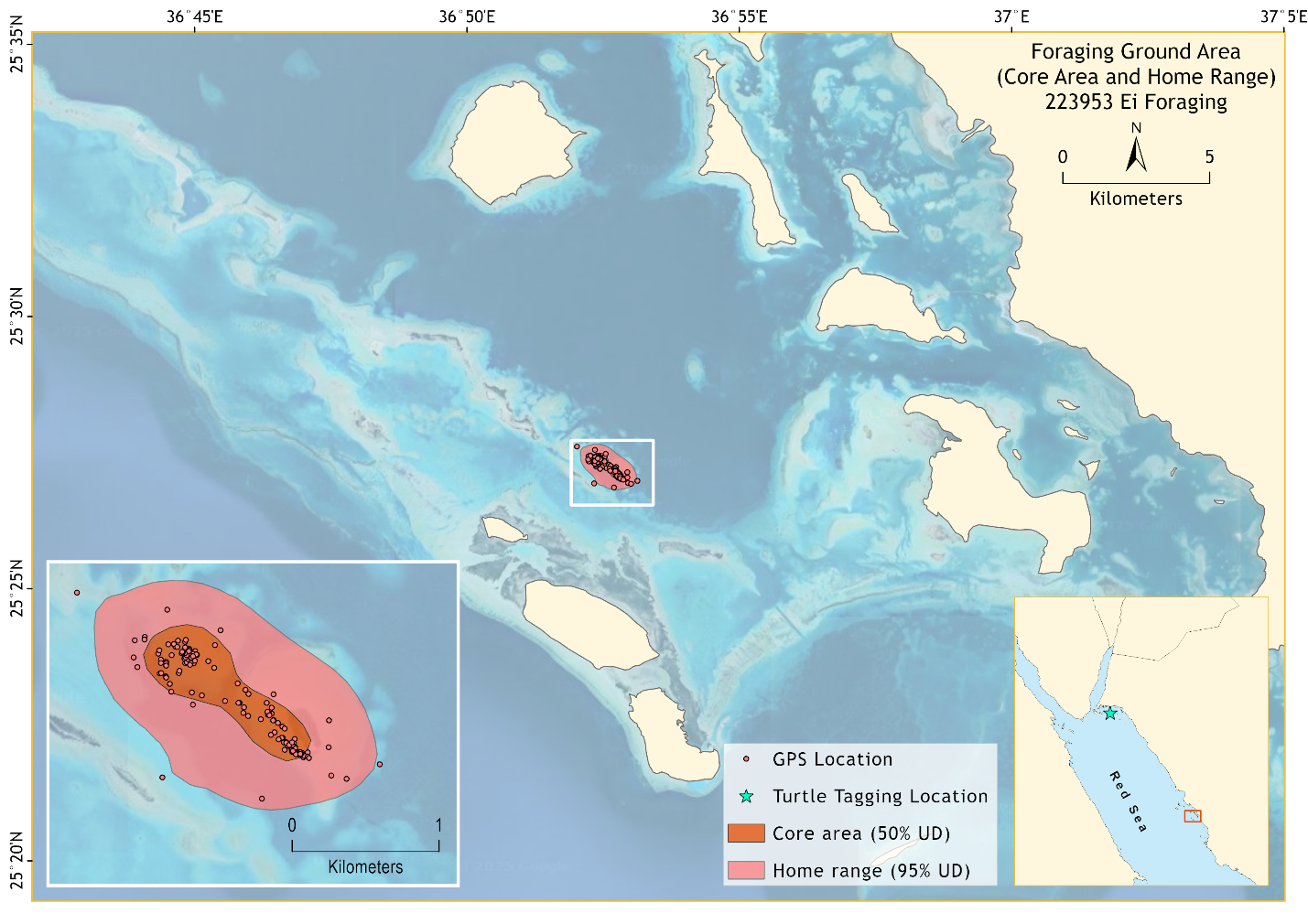

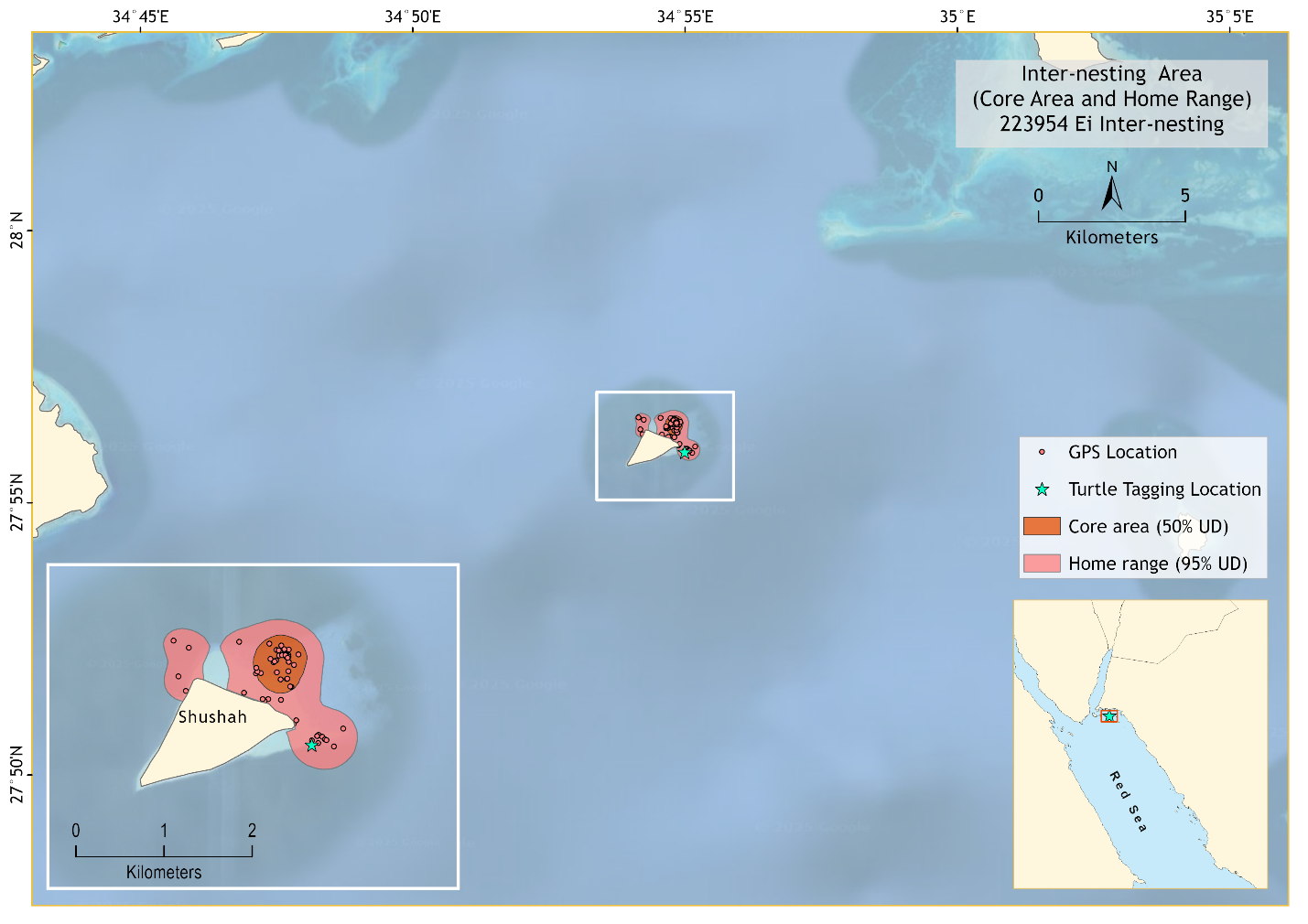

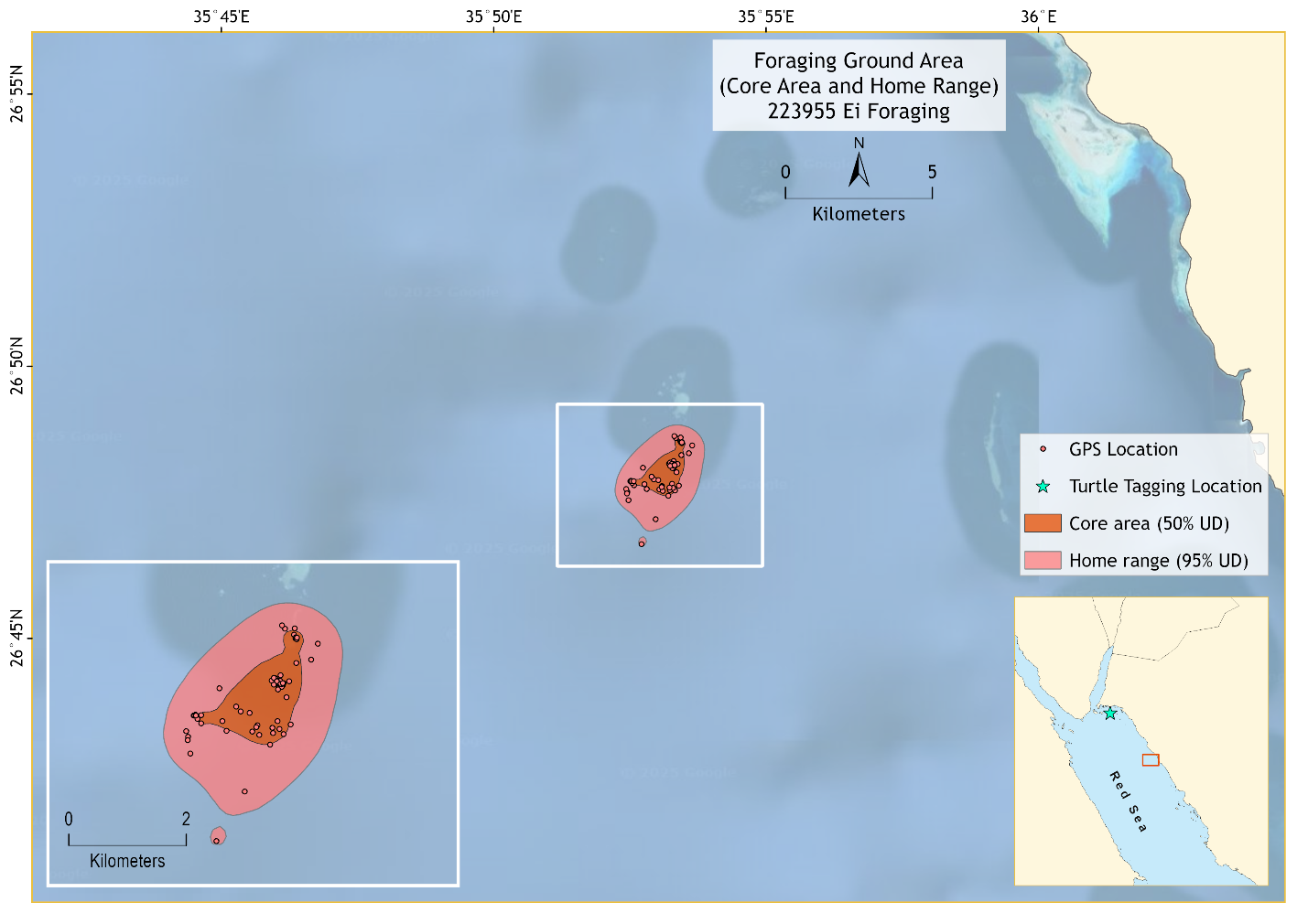

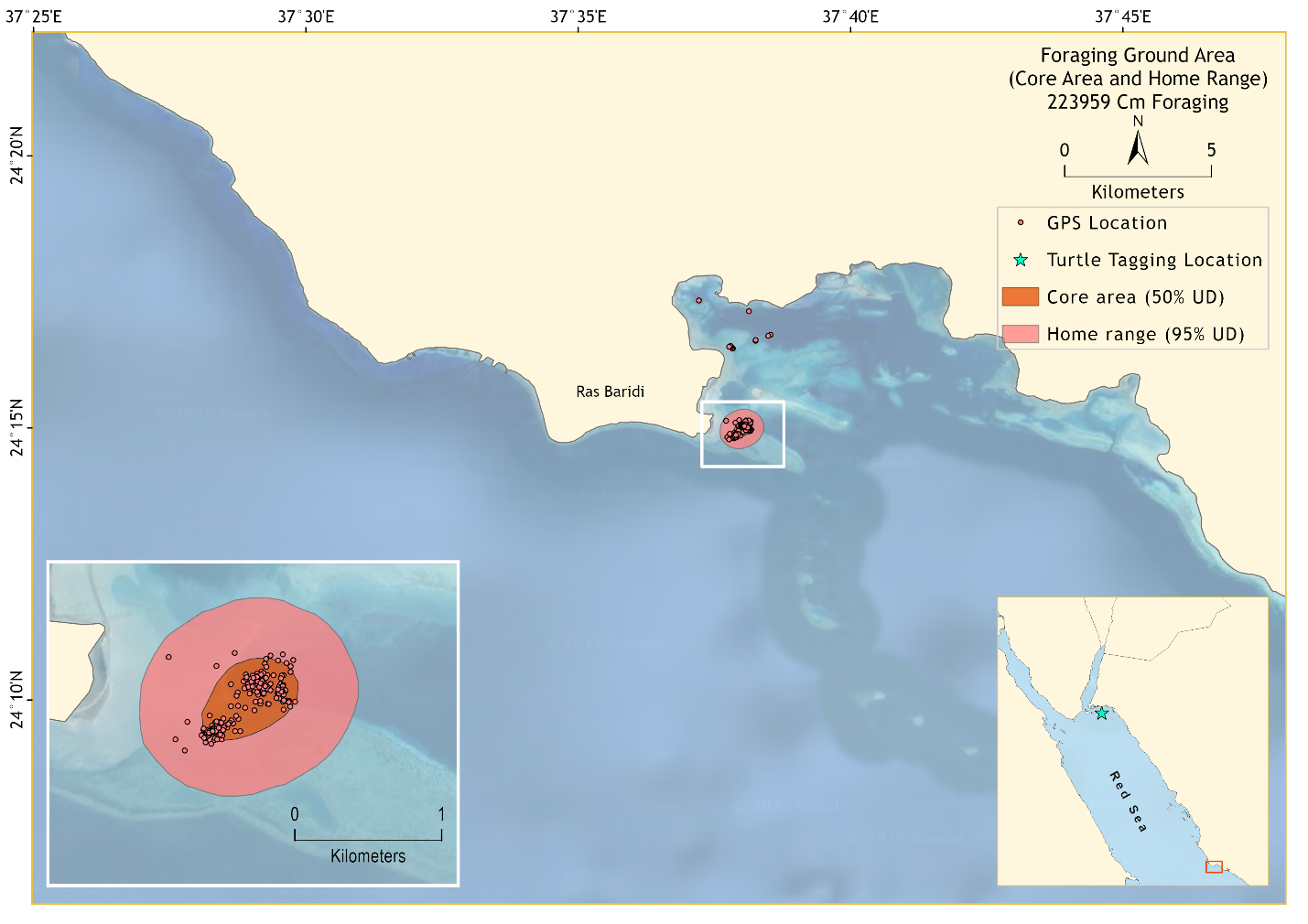

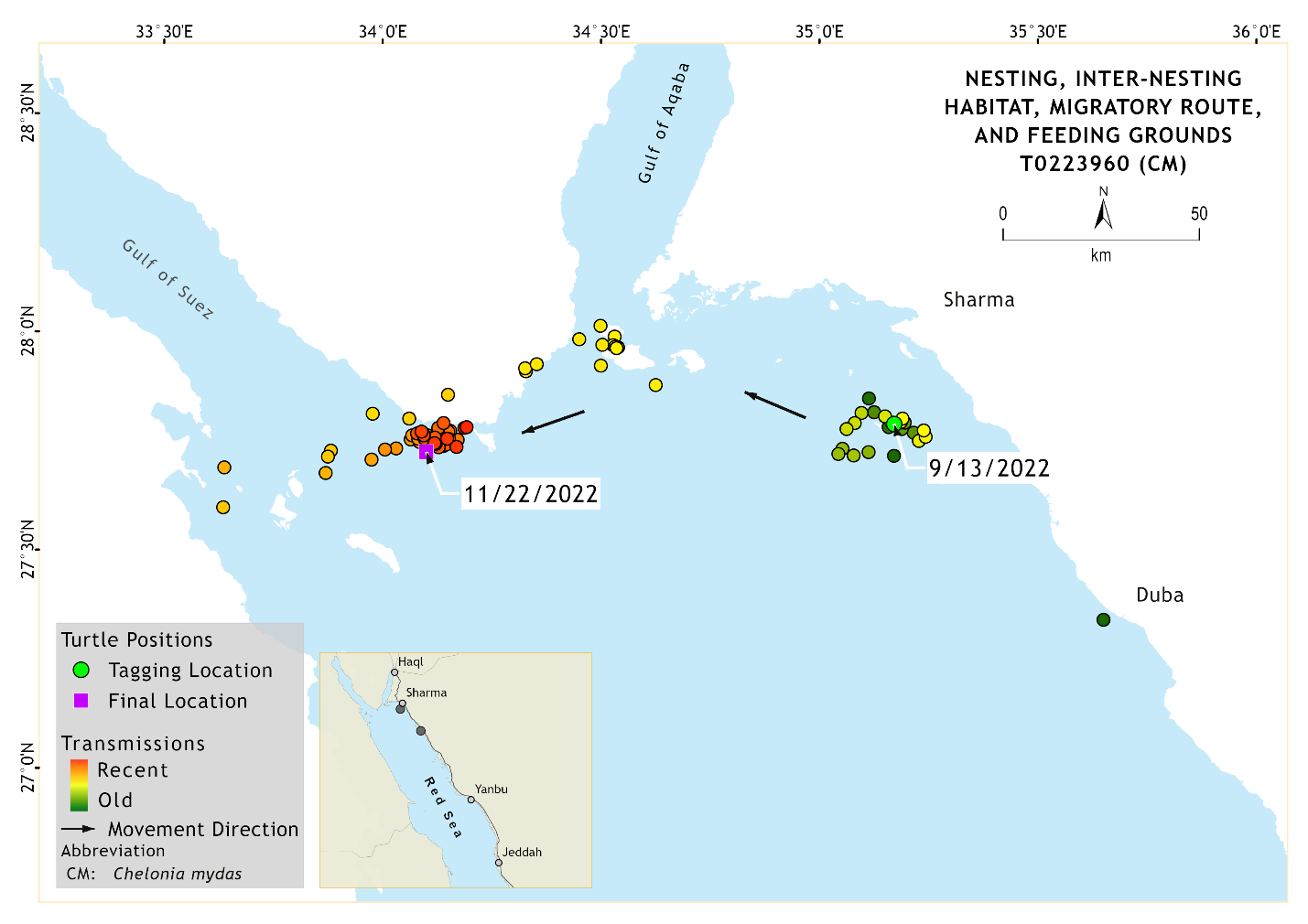

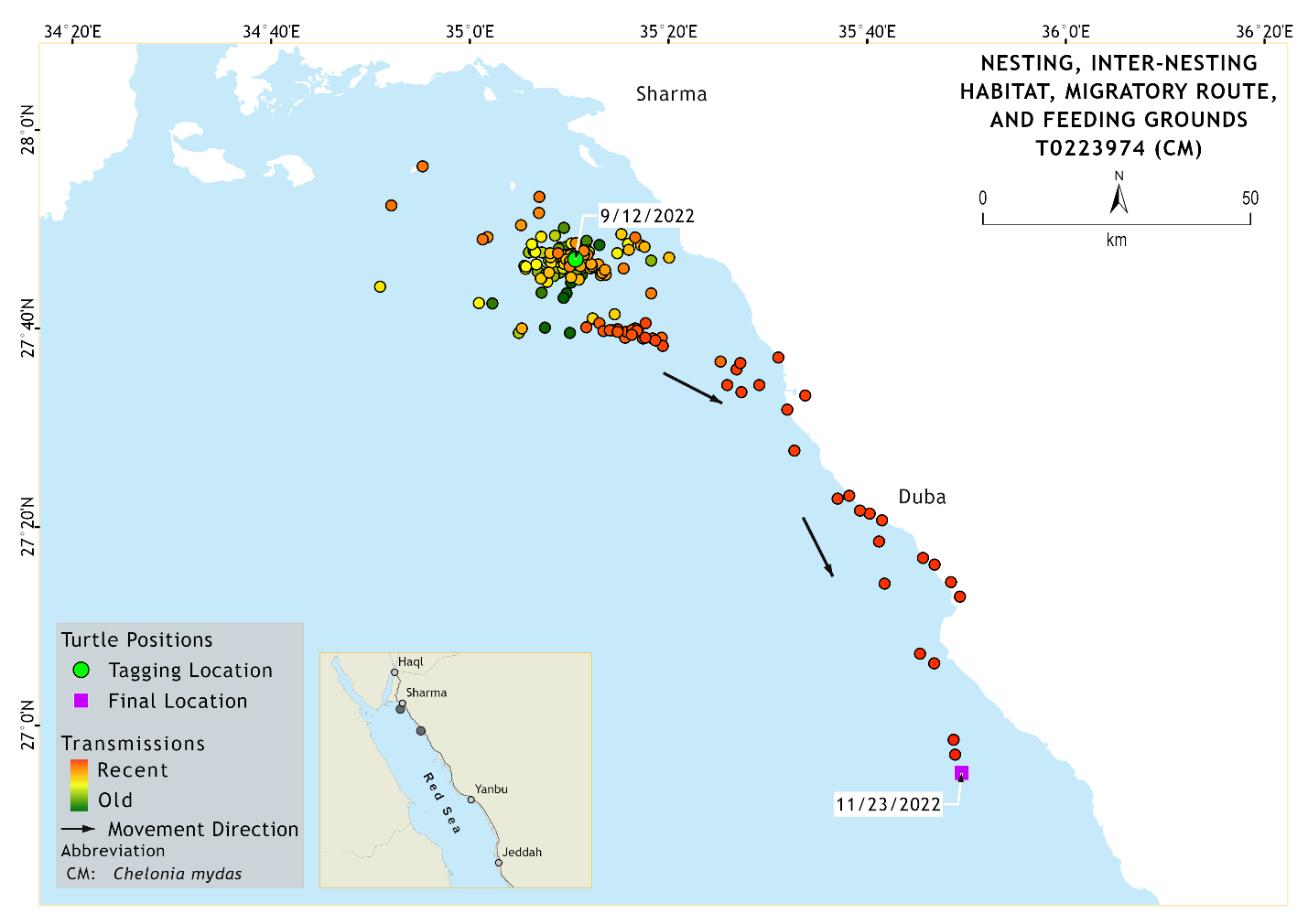

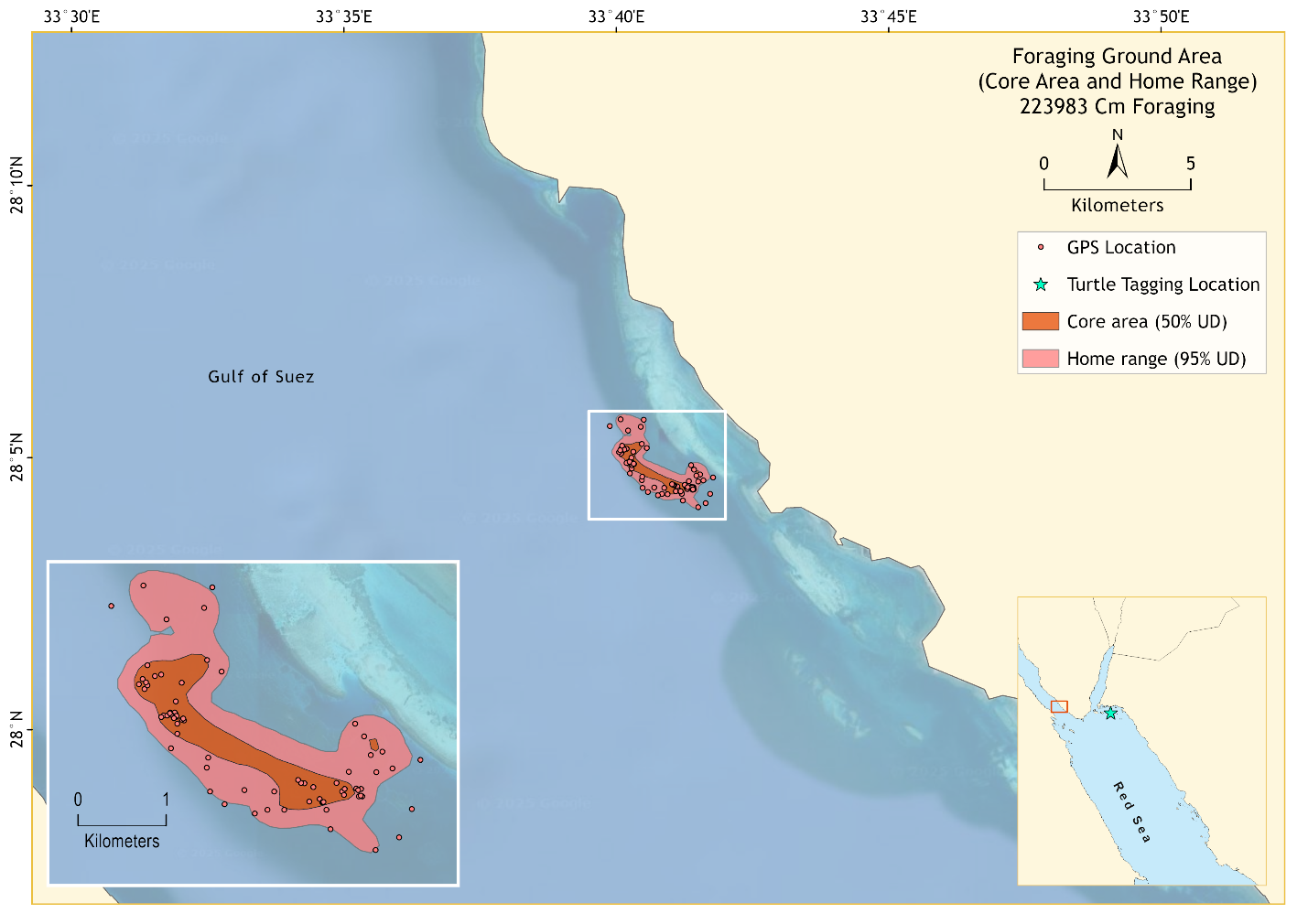


**Table 1. Duty Cycle Configuration of Lotek FastGPS Argos Tags Used in This Study**

| **Phase** | **Start Day** | **End Day** | **GPS Fix Interval (min)** | **Argos Uplink Limit (messages/day)** | **Retry Settings** |
| --- | --- | --- | --- | --- | --- |
| 0 | 0 | 60 | 15 | 400 | Up to 3 retries; 1-hour retry interval |
| 1 | 61 | End of battery life | 30 | 400 | Up to 3 retries; 1-hour retry interval |
