# Supplementary material for: Post-breeding dispersal of nesting marine turtles from the NEOM Islands, Saudi Arabia
Source: Sci Rep. 2025 Dec 23;16:1695. doi: 10.1038/s41598-025-31237-1 (PMC12800070; doi:10.1038/s41598-025-31237-1)
Supplement: Supplementary file 2 — Supplementary Material 2 [file 41598_2025_31237_MOESM2_ESM.docx]

Supplementary Materials for the article:

**Dispersal of nesting marine turtles from the NEOM Islands, Saudi Arabia, after breeding**

Hector Barrios-Garrido^1^; Abdulrazaq Alatawi^1^; Mishari Alghrair^2^; Abdulaziz Alkaboor^1^; Enjey Ghazzawi^2^; Abdulqader Khamis^2^; Brett Lyons^2^; Paul Marshall^2^; Abhishekh Palaparambil Vijaya^1^; August Santillan^1^; Deni Porej^2^; Winston Cowie^2^; Ricardo O. Ramalho^1^

1 KAUST Beacon Development; KAUST Innovation; King Abdullah University of Science and Technology (KAUST). Thuwal, Makkah, 23955; Kingdom of Saudi Arabia.

2 NEOM. Sharma 49631; Kingdom of Saudi Arabia.

Corresponding author: [hector.barrios@kaust.edu.sa](mailto:hector.barrios@kaust.edu.sa)

This supplementary material contains maps of the (i) inter-nesting habitat use), (ii) movement direction maps; and (iii) foraging sites, including their home ranges (UD 95%) and core areas (UD 50%) from the turtles tagged in this study.

Data transmission varied among individuals, in terms of locations, accuracy, and due to technical limitations. Hence, Table S1 summarizes the maps produced discriminated by individual to inform the reader on what elements are available per tagged animal.

Section (i) presents the spatial distribution and inter-nesting habitat maps for three hawksbill turtles (PTTs: 0223945, 0223950, and 0223954).

This file contains maps illustrating (ii) movement patters of tagged turtles, highlighting the tagging and last transmitted locations. Transmission points are color-coded, with older locations in yellow and recent locations of the transmitted fixes in orange. The black arrows represent the turtle movement direction (not necessarily the migratory route). It is represented on that way due to limitations in the data transmitted by the PTTs between tagging locations and final (potential feeding) location.

Section (iii) details the home ranges and core habitats for up to nine of the identified foraging grounds individually; three greens (PTTs: 0223946, 0223959, 0223983) and six hawksbills (PTTs: 0223945, 0223949, 0223950, 0223952, 0223953, 0223055).

An additional table with the duty cycle configuration used is included here (Table S2).

Table S1. Summary table of data transmitted and mapped on this supplementary material document

| Species | Turtle ID (Flipper Tags) | PTT Code | Inter-nesting Habitat | Movement Patter Map | Feeding Ground Habitat  (Home Range and Core Area) | Tracked Days |
| --- | --- | --- | --- | --- | --- | --- |
| *Chelonia mydas* | RS-0457  RS-0456 | 0223959 | No | No | Yes | 440 |
|  | RS-0458  RS-0461 | 0223946 | No | No | Yes | 95 |
|  | RS-0464  RS-0466 | 0223947 | No | Yes | No | 31 |
|  | RS-0467  RS-0468 | 0223974 | No | Yes | No | 84 |
|  | RS-0469  RS-0470 | 0223960 | No | Yes | No | 187 |
|  | RS-0471  RS-0472 | 0223983 | No | No | Yes | 314 |
| *Eretmochelys imbricata* | RS-0462  RS-0463 | 0223944 | No | Yes | No | 43 |
|  | RS-0473  RS-0474 | 0223943 | No | No | No | 12 |
|  | RS-0475 RS-0476 | 0223945 | Yes | No | Yes | 162 |
|  | RS-0477  RS-0478 | 0223948 | No | Yes | No | 31 |
|  | RS-0479  RS-0480 | 0223949 | No | No | Yes | 157 |
|  | RS-0481  RS-0482 | 0223950 | Yes | No | Yes | 157 |
|  | RS-0483  RS-0484 | 0223951 | No | Yes | No | 9 |
|  | RS-0485  RS-0486 | 0223952 | No | No | Yes | 155 |
|  | RS-0487  RS-0488 | 0223953 | No | No | Yes | 155 |
|  | RS-0489  RS-0490 | 0223954 | Yes | No | No | 4 |
|  | RS-0491  RS-0492 | 0223955 | No | No | Yes | 153 |

1. *Inter-Nesting Habitat Use (UDs) Maps*

**Figure S1. PTT-0223945 Turtle Inter-Nesting Habitat Use: Core Area and Home Range (Utilization Distribution – UDs)**

***Eretmochelys imbricata***


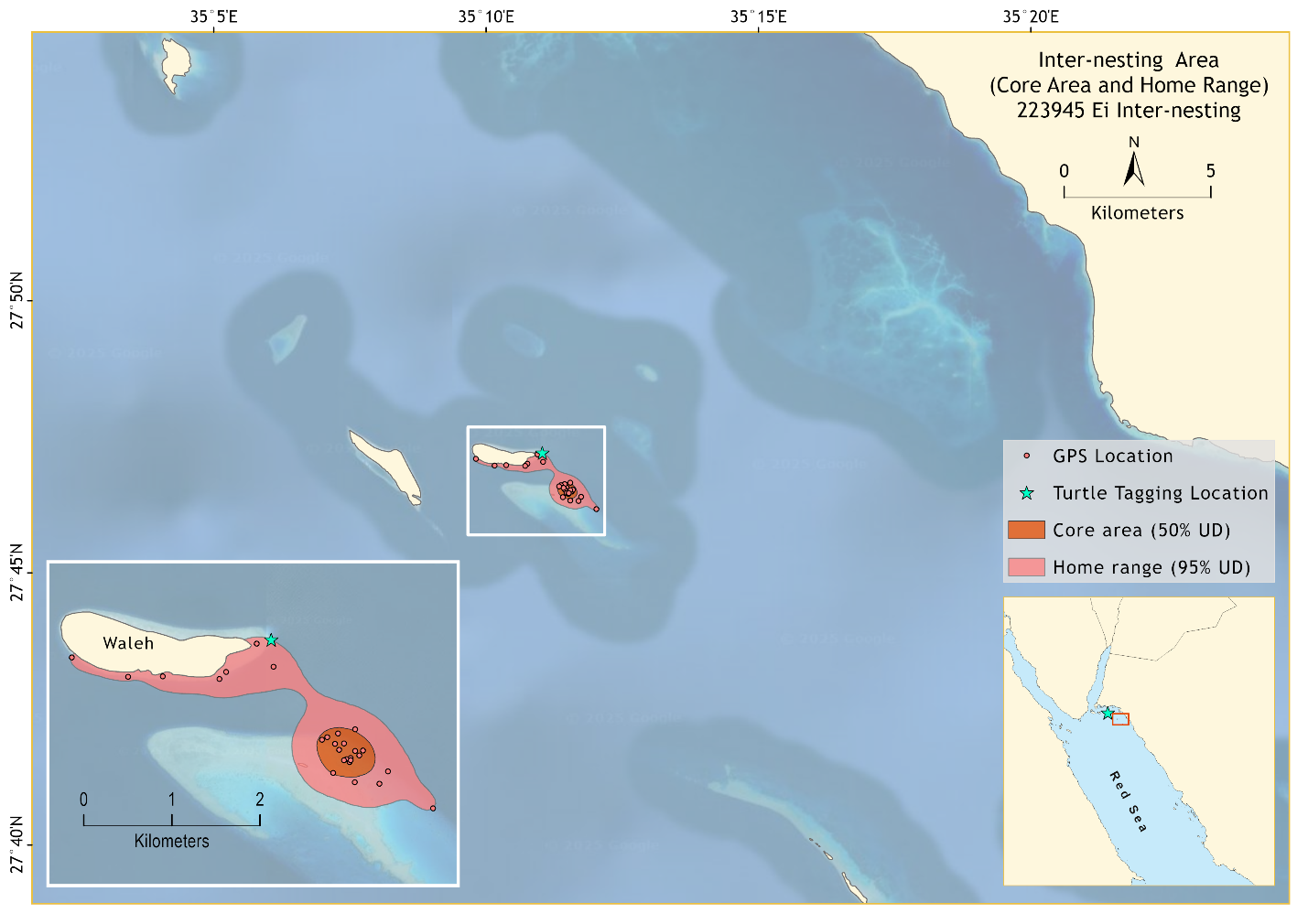


**Figure S2. PTT-0223950 Turtle Inter-Nesting Habitat Use: Core Area and Home Range (Utilization Distribution – UDs)**

***Eretmochelys imbricata***


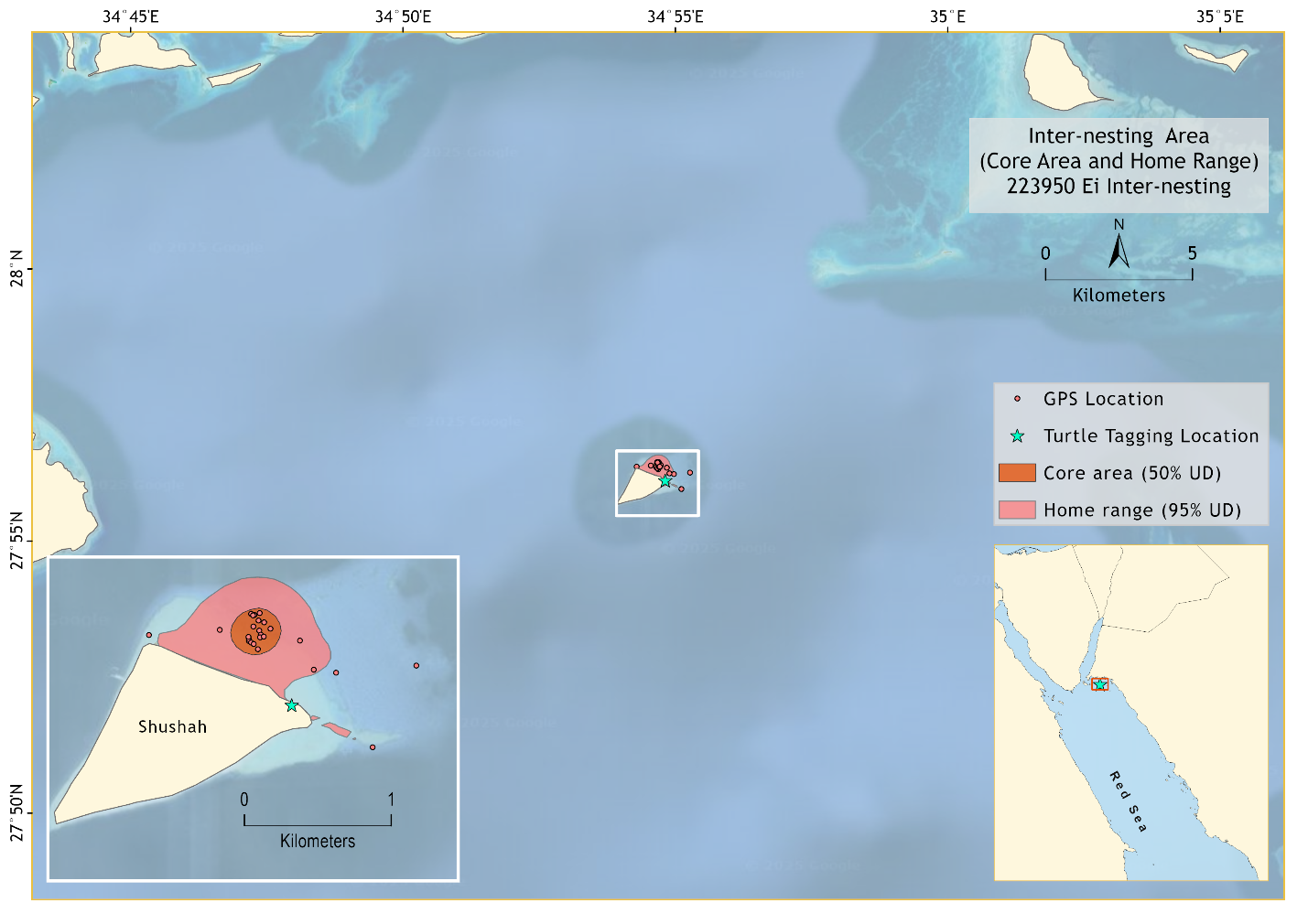


**Figure S3. PTT-0223954 Turtle Inter-Nesting Habitat Use: Core Area and Home Range (Utilization Distribution – UDs)**

***Eretmochelys imbricata***


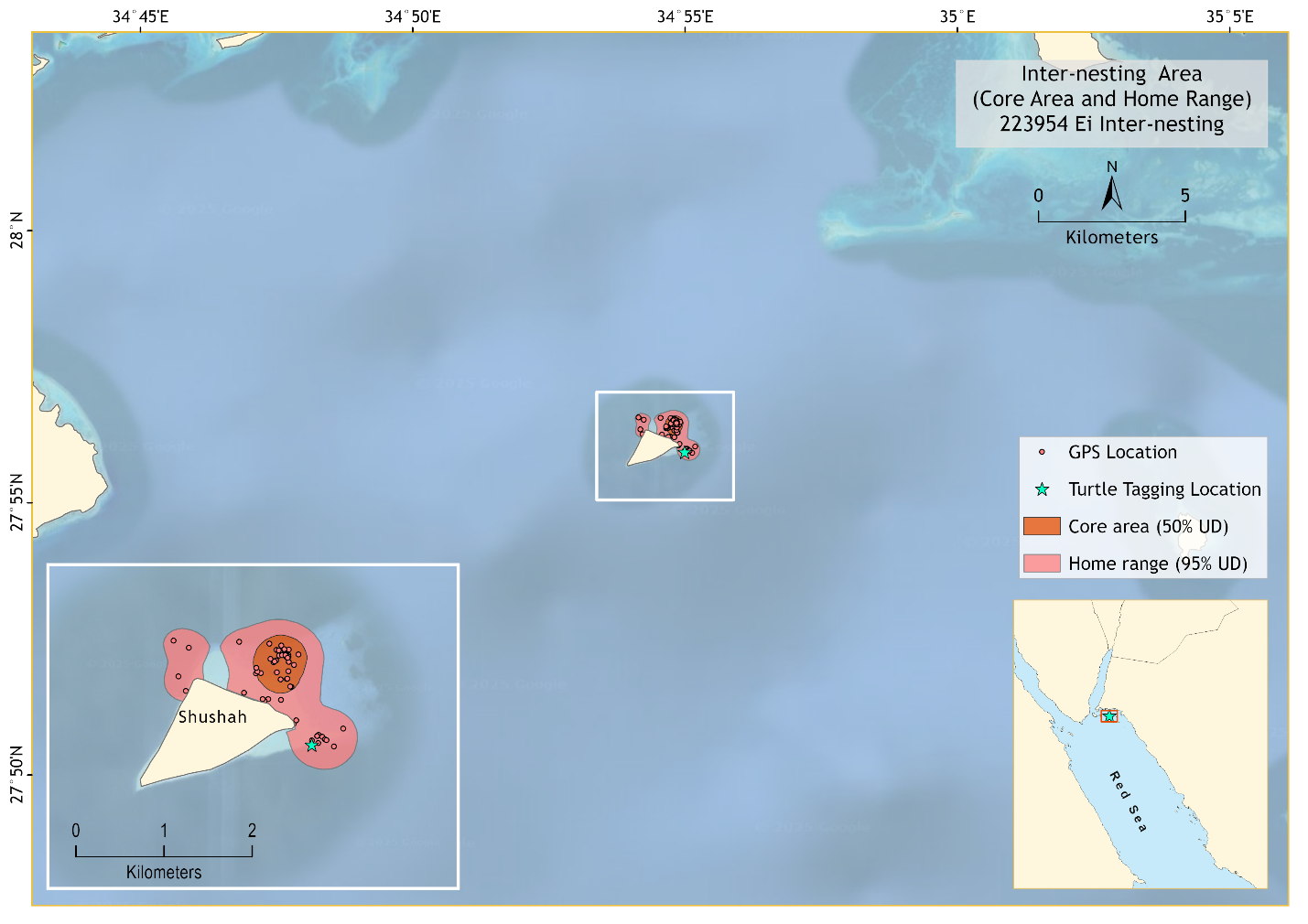


1. *Movement Direction Maps*

**Figure S4. PTT-0223947 Turtle Movement Direction representation based on Known Tagging Location and Final Transmitted Location**

***Chelonia mydas***


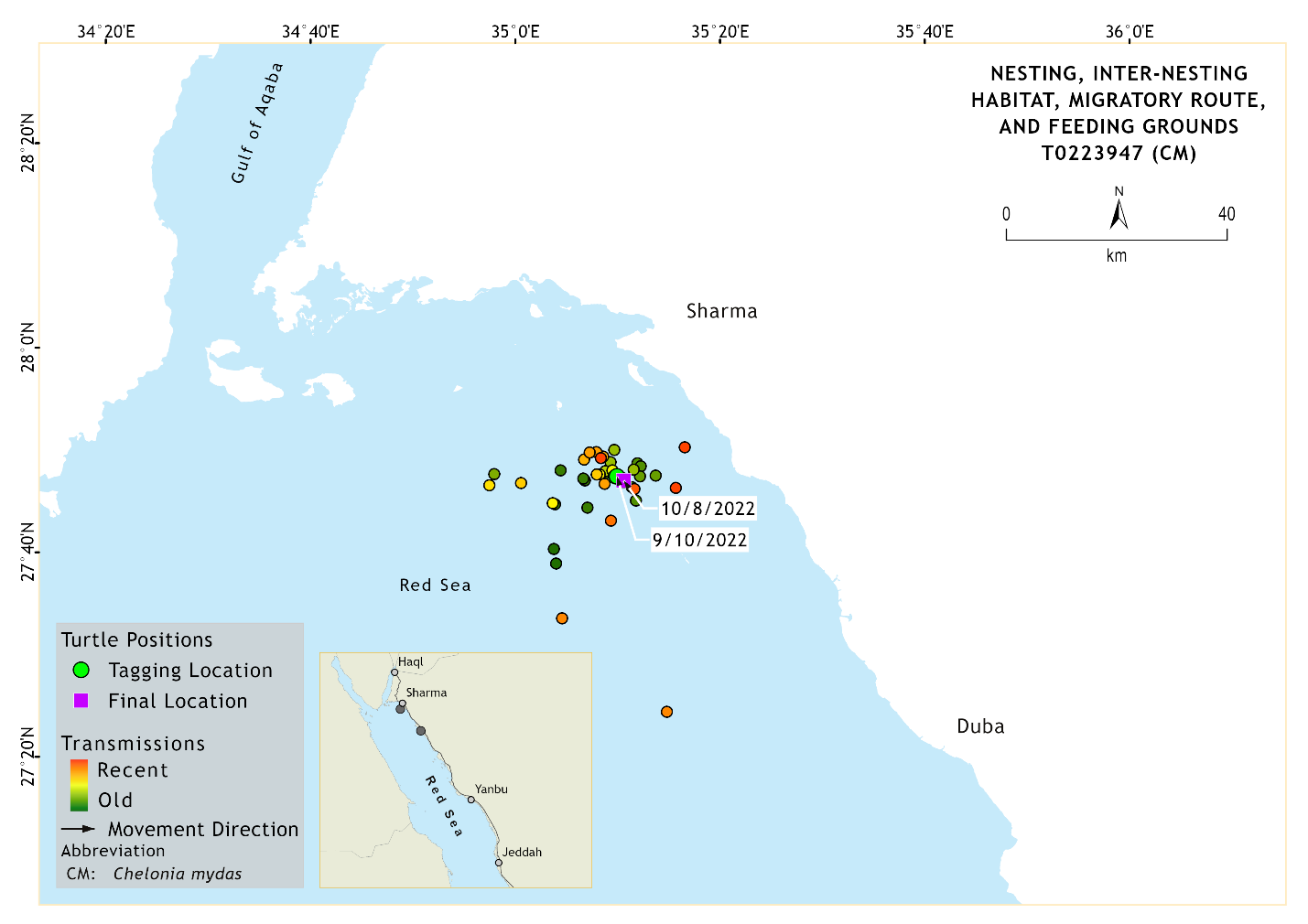


**Figure S5. PTT-0223974 Turtle Movement Direction representation based on Known Tagging Location and Final Transmitted Location**

***Chelonia mydas***


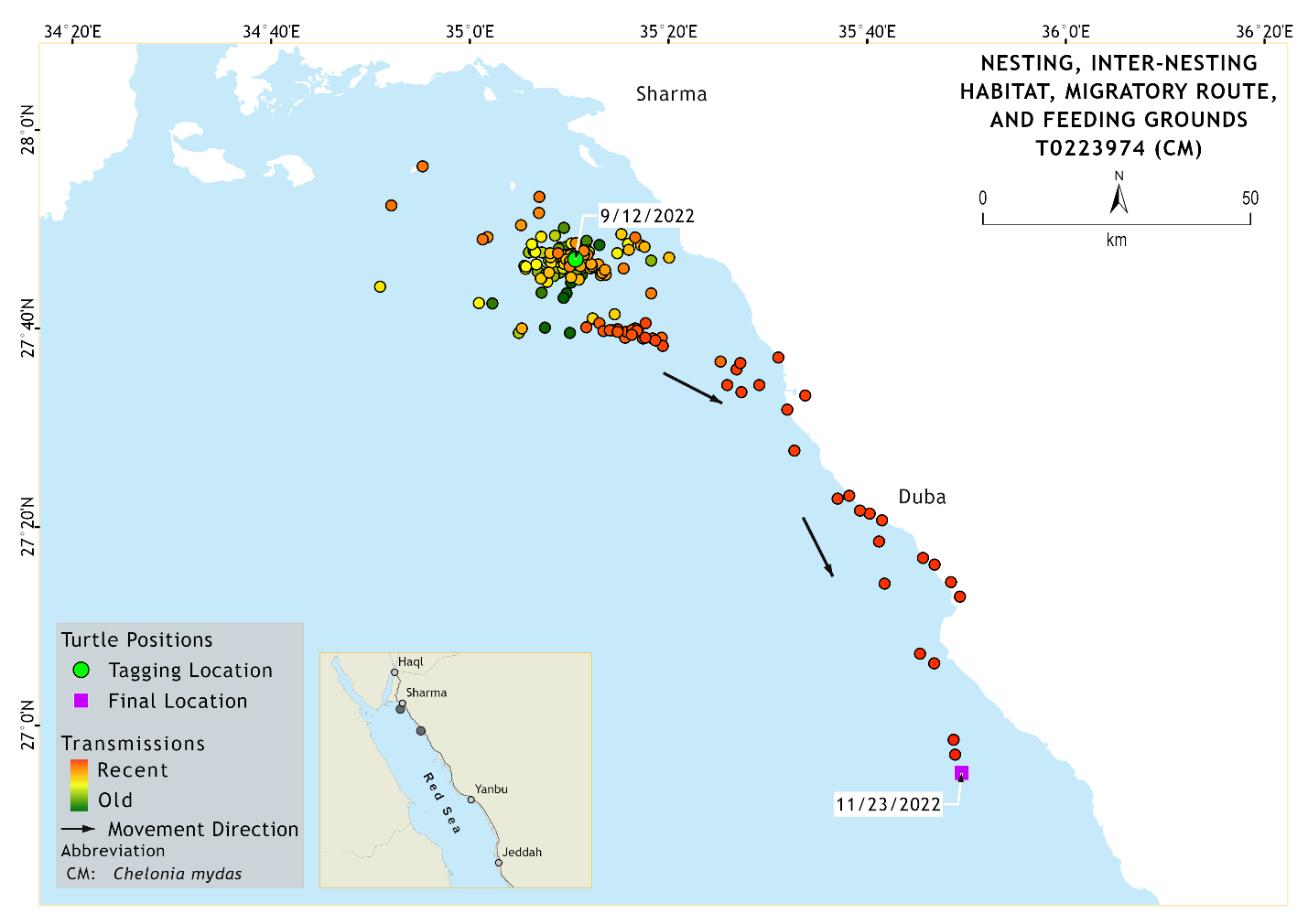


**Figure S6. PTT-0223960 Turtle Movement Direction representation based on Known Tagging Location and Final Transmitted Location**

***Chelonia mydas***


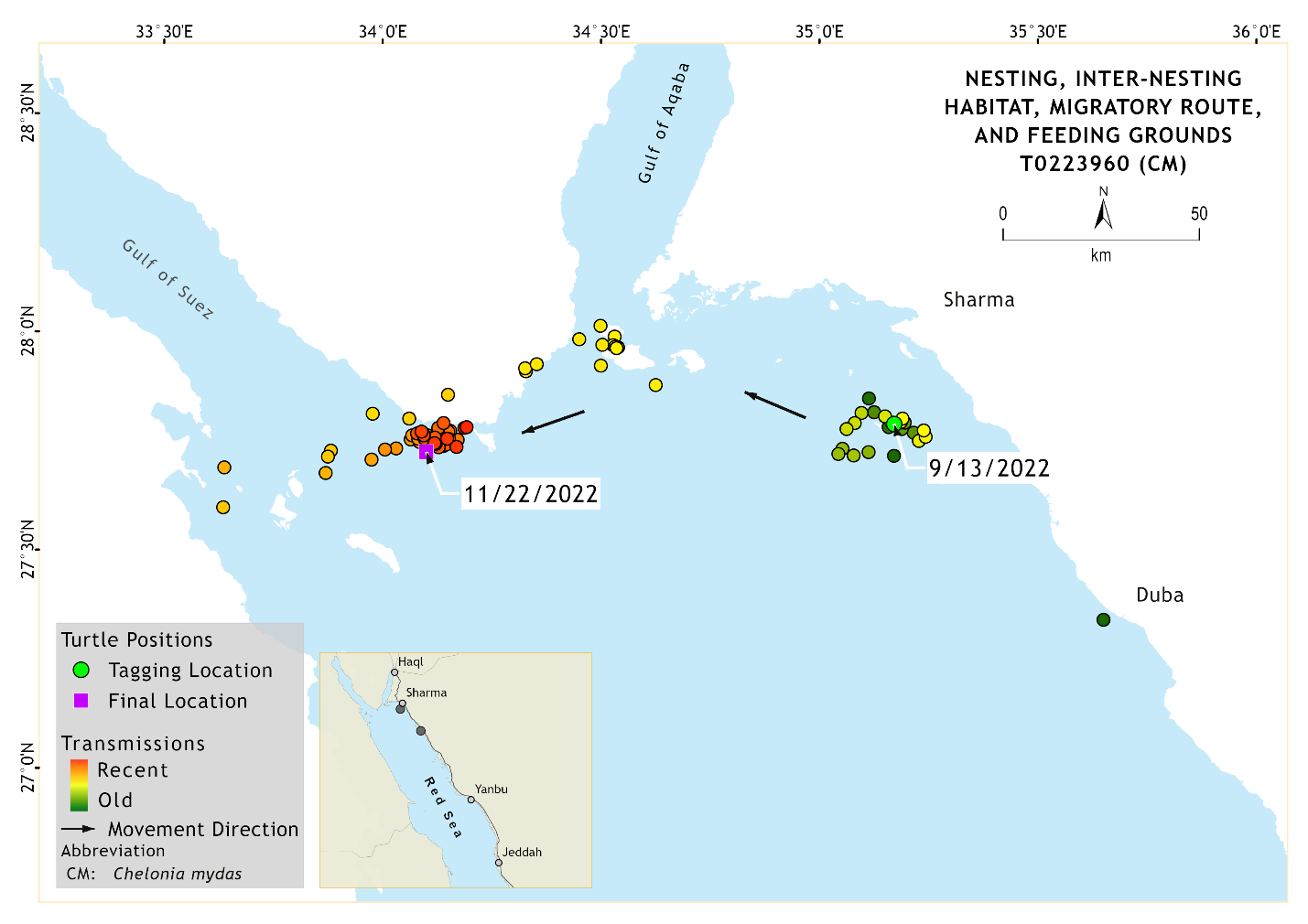


**Figure S7. PTT-0223944 Turtle Movement Direction representation based on Known Tagging Location and Final Transmitted Location**

***Eretmochelys imbricata***


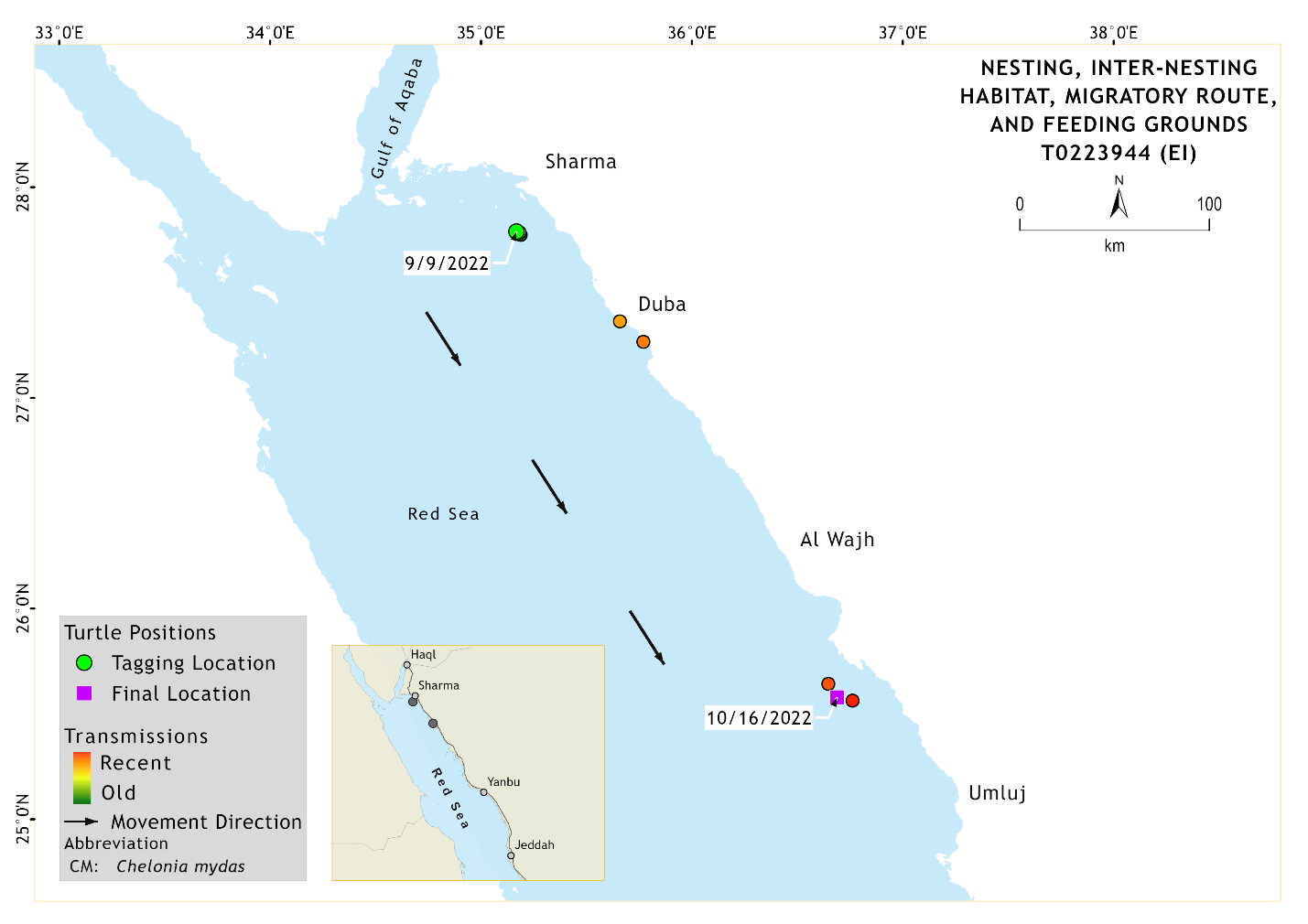


**Figure S8. PTT-0223948 Turtle Movement Direction representation based on Known Tagging Location and Final Transmitted Location**

***Eretmochelys imbricata***
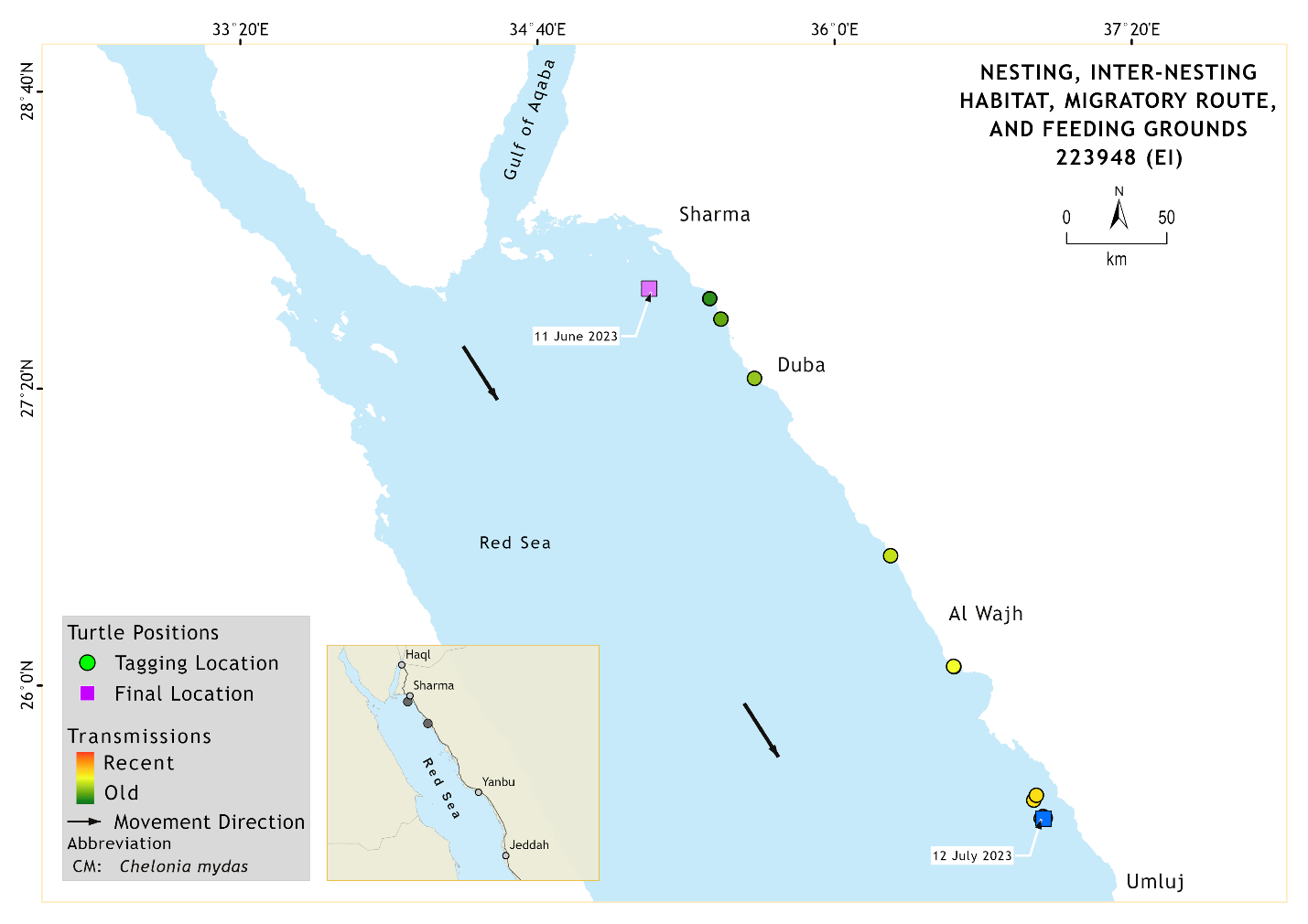


**Figure S9. PTT-0223951 Turtle Movement Direction representation based on Known Tagging Location and Final Transmitted Location**

***Eretmochelys imbricata***


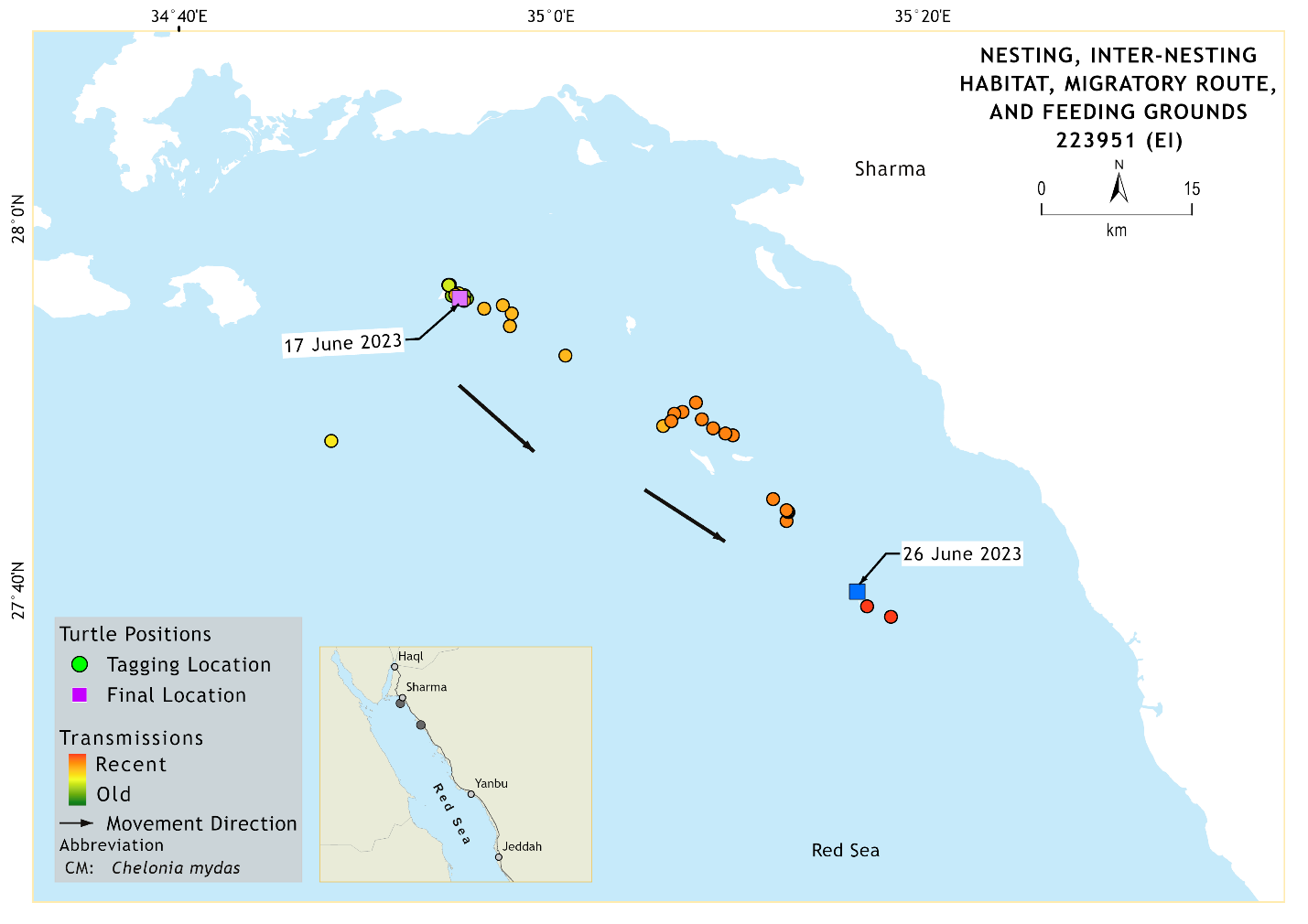


1. *Foraging Habitat Use – Maps*

**Figure S10. PTT-0223959 Turtle Foraging Habitat Use: Core Area and Home Range (Utilization Distribution – UDs)**

***Chelonia mydas***


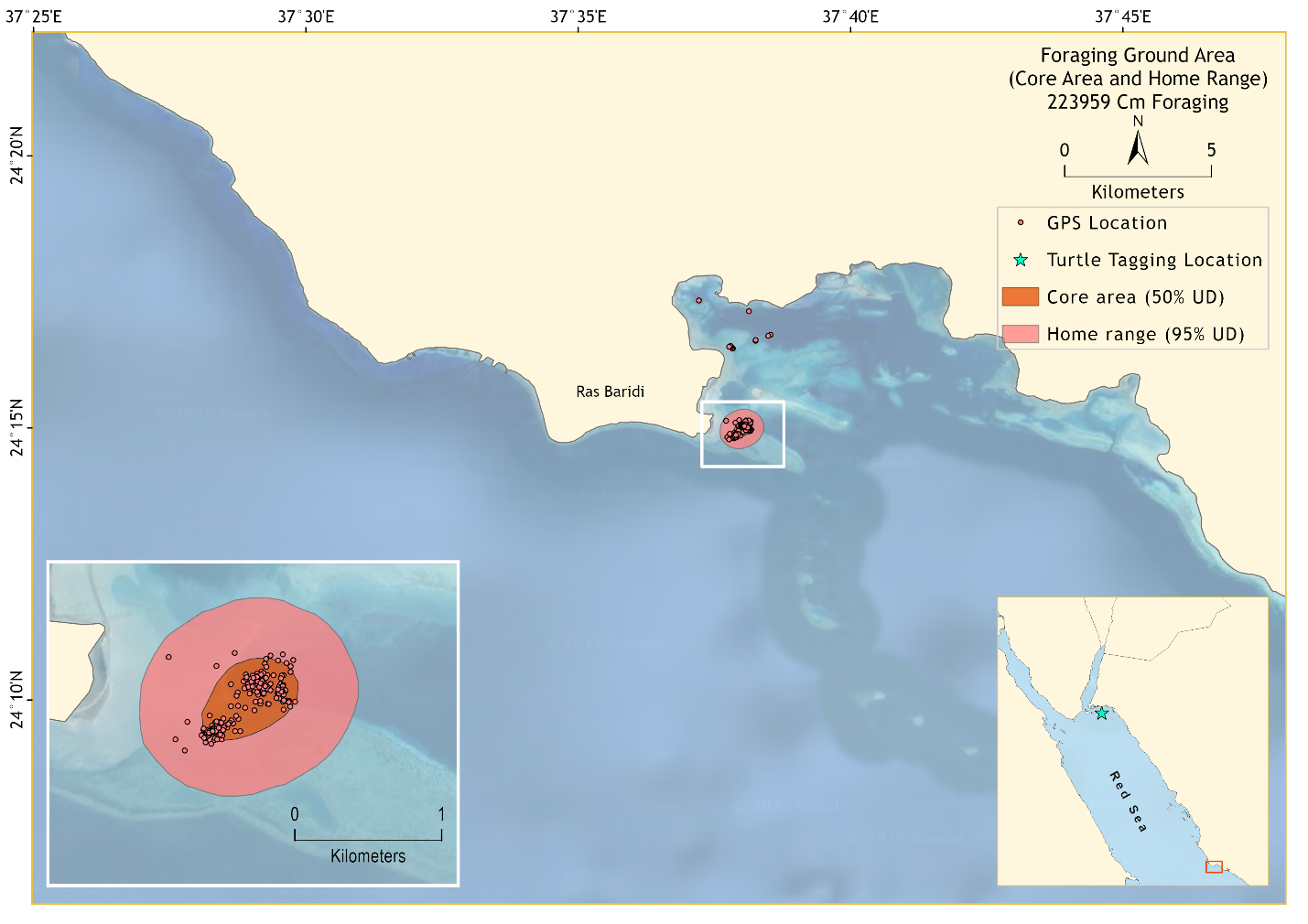


**Figure S11. PTT-0223946 Turtle Foraging Habitat Use: Core Area and Home Range (Utilization Distribution – UDs)**

***Chelonia mydas***


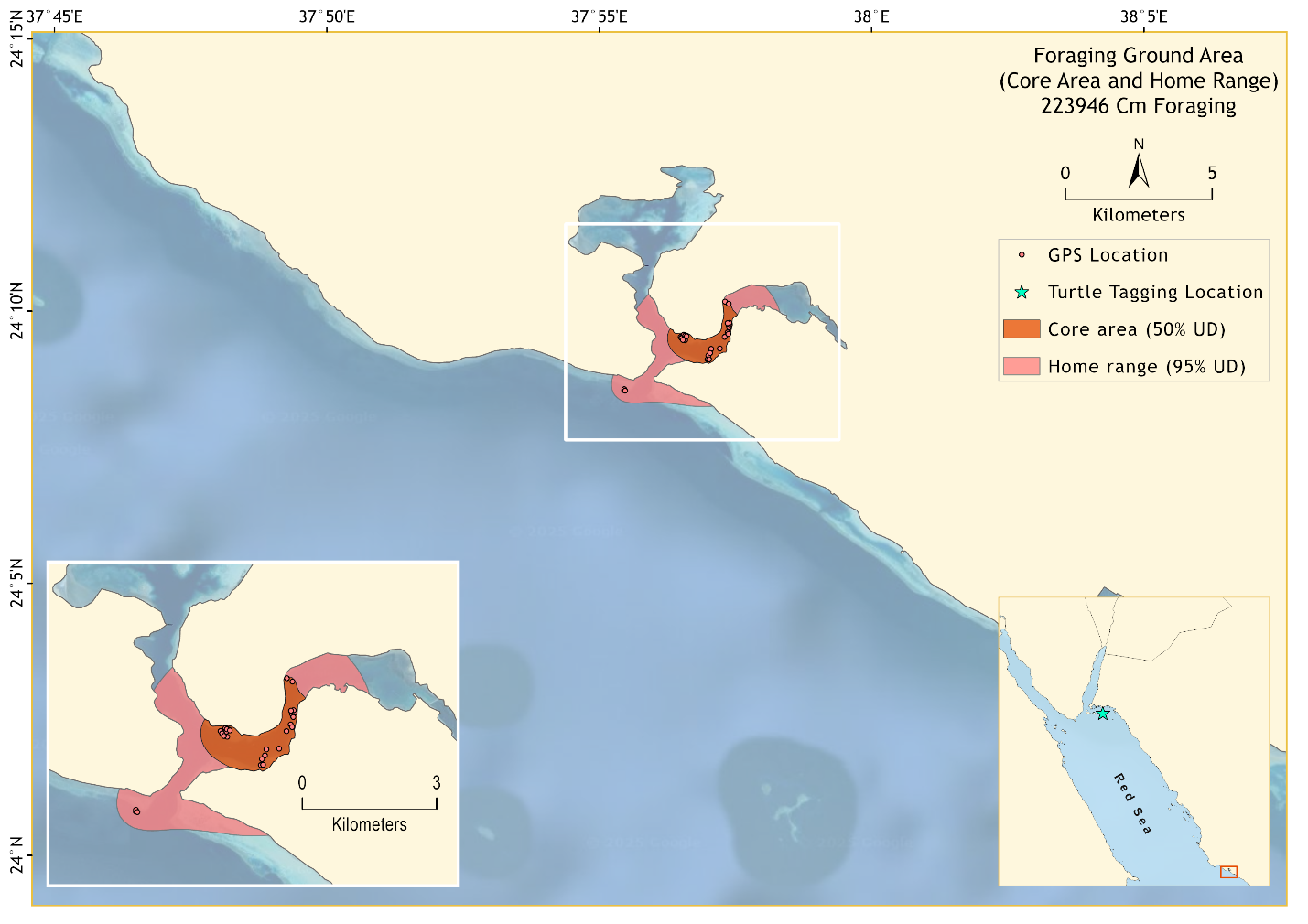


**Figure S12. PTT-0223983 Turtle Foraging Habitat Use: Core Area and Home Range (Utilization Distribution – UDs)**

***Chelonia mydas***


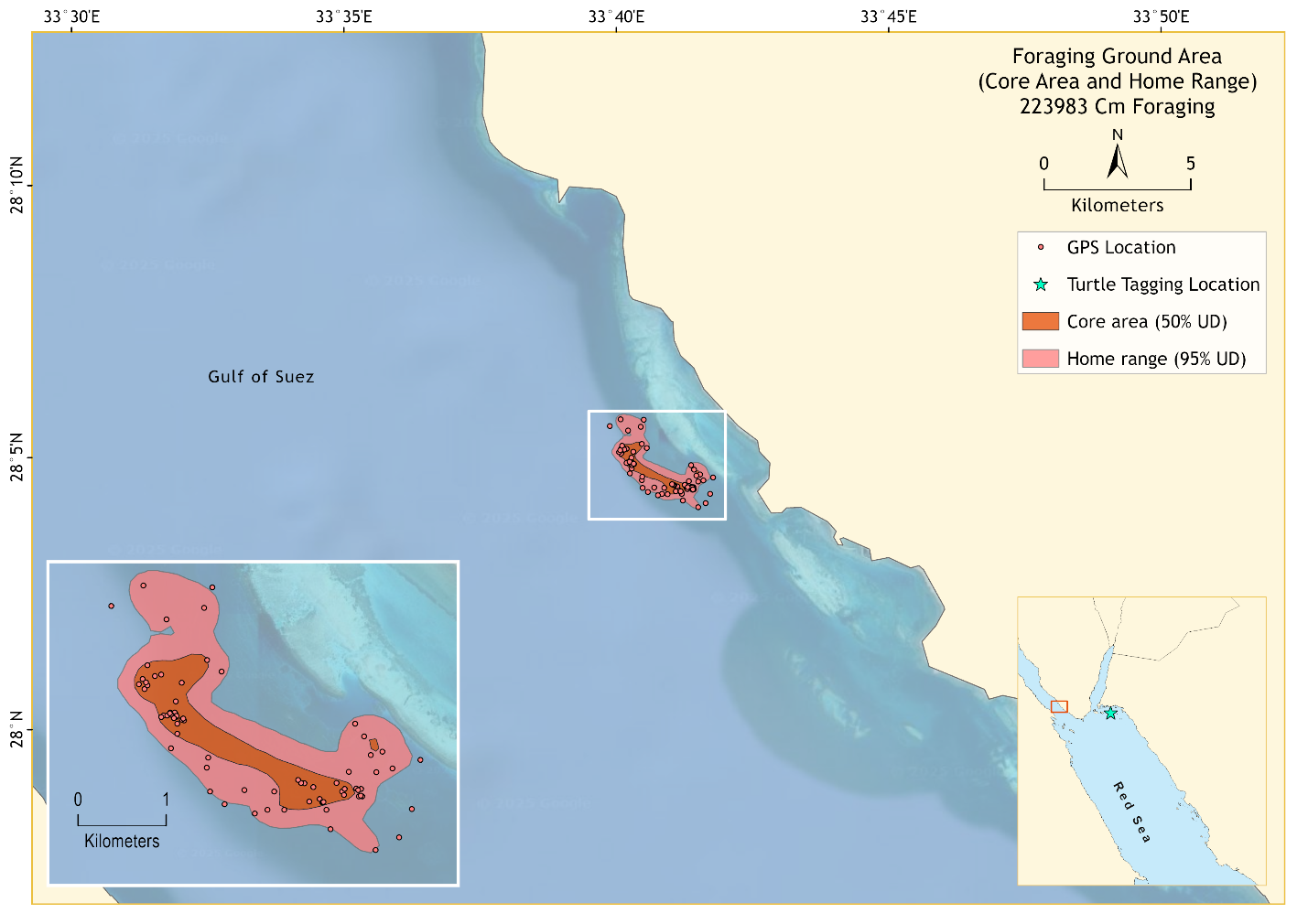


**Figure S13. PTT-0223945 Turtle Foraging Habitat Use: Core Area and Home Range (Utilization Distribution – UDs)**

***Eretmochelys imbricata***


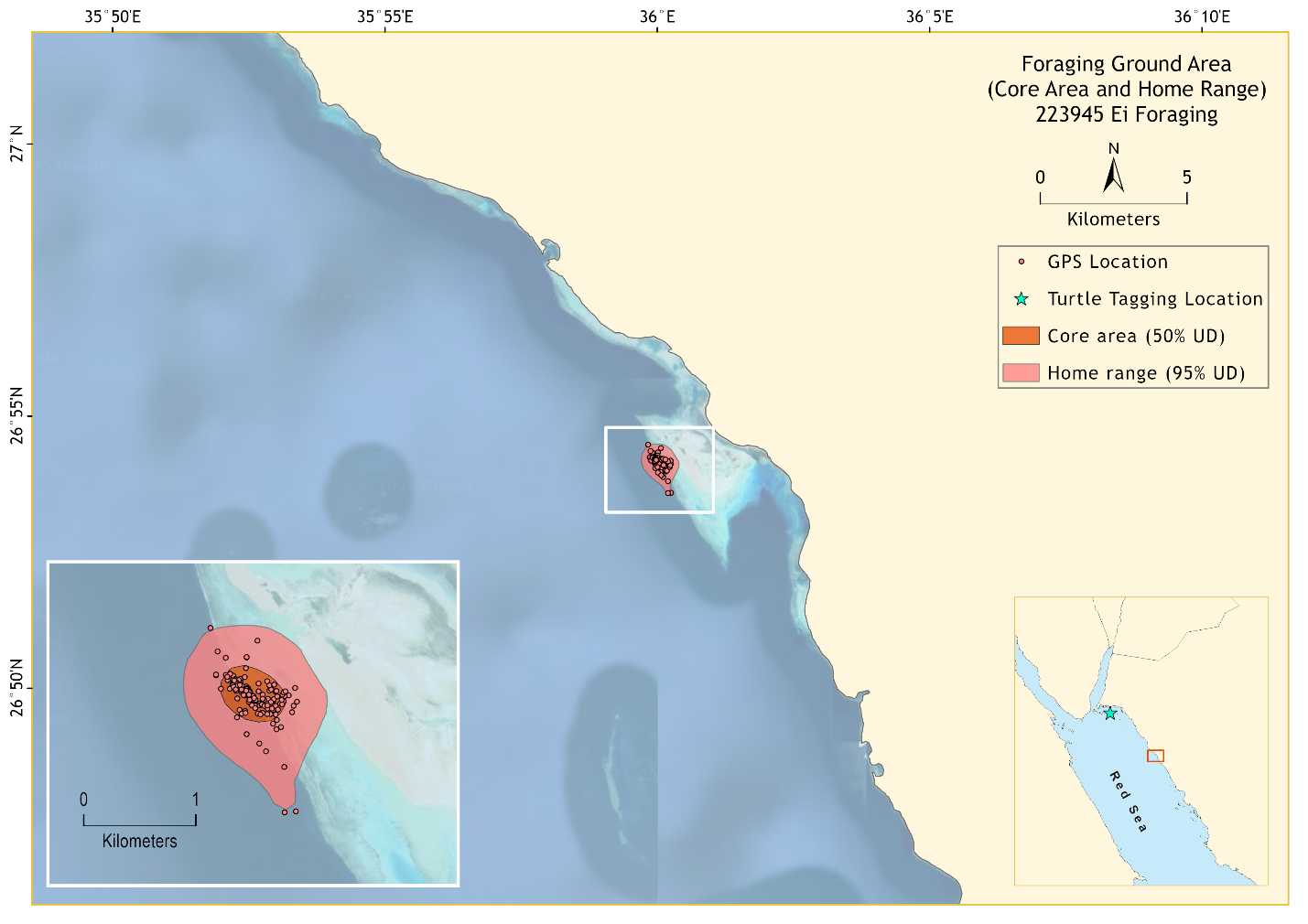


**Figure S14. PTT-0223949 Turtle Foraging Habitat Use: Core Area and Home Range (Utilization Distribution – UDs)**

***Eretmochelys imbricata***
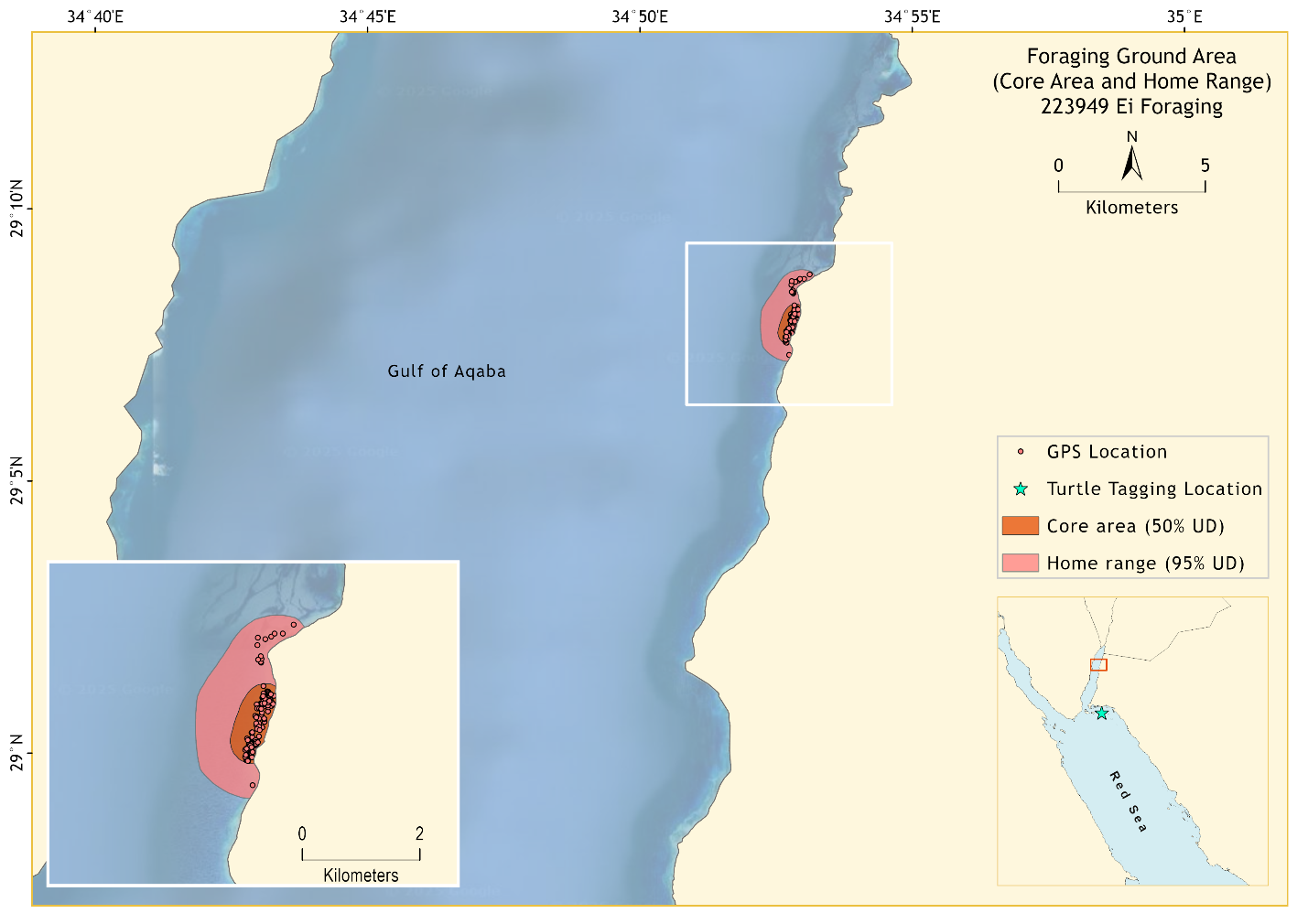


**Figure S15. PTT-0223950 Turtle Foraging Habitat Use: Core Area and Home Range (Utilization Distribution – UDs)**

***Eretmochelys imbricata***


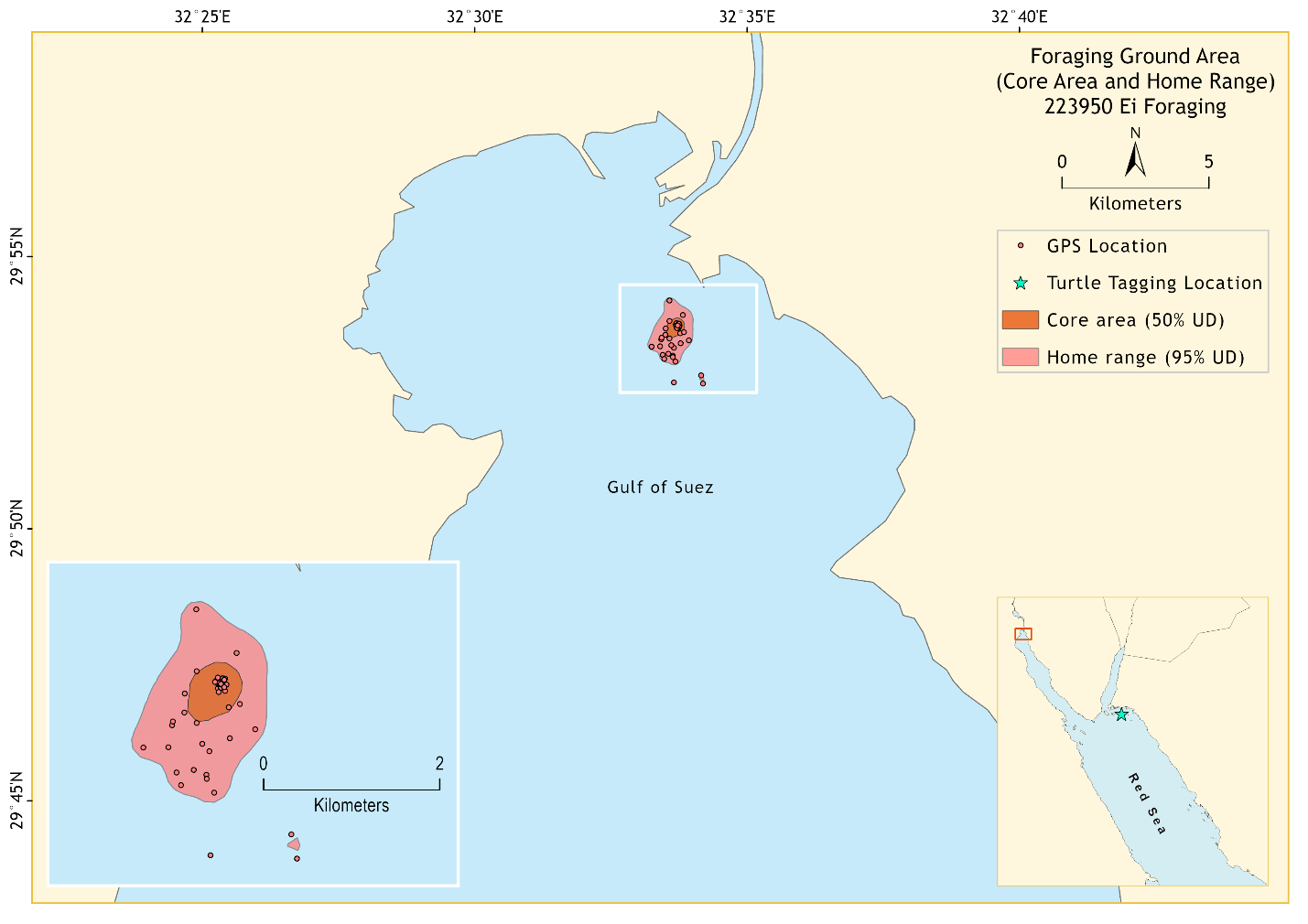


**Figure S16. PTT-0223952 Turtle Foraging Habitat Use: Core Area and Home Range (Utilization Distribution – UDs)**

***Eretmochelys imbricata***


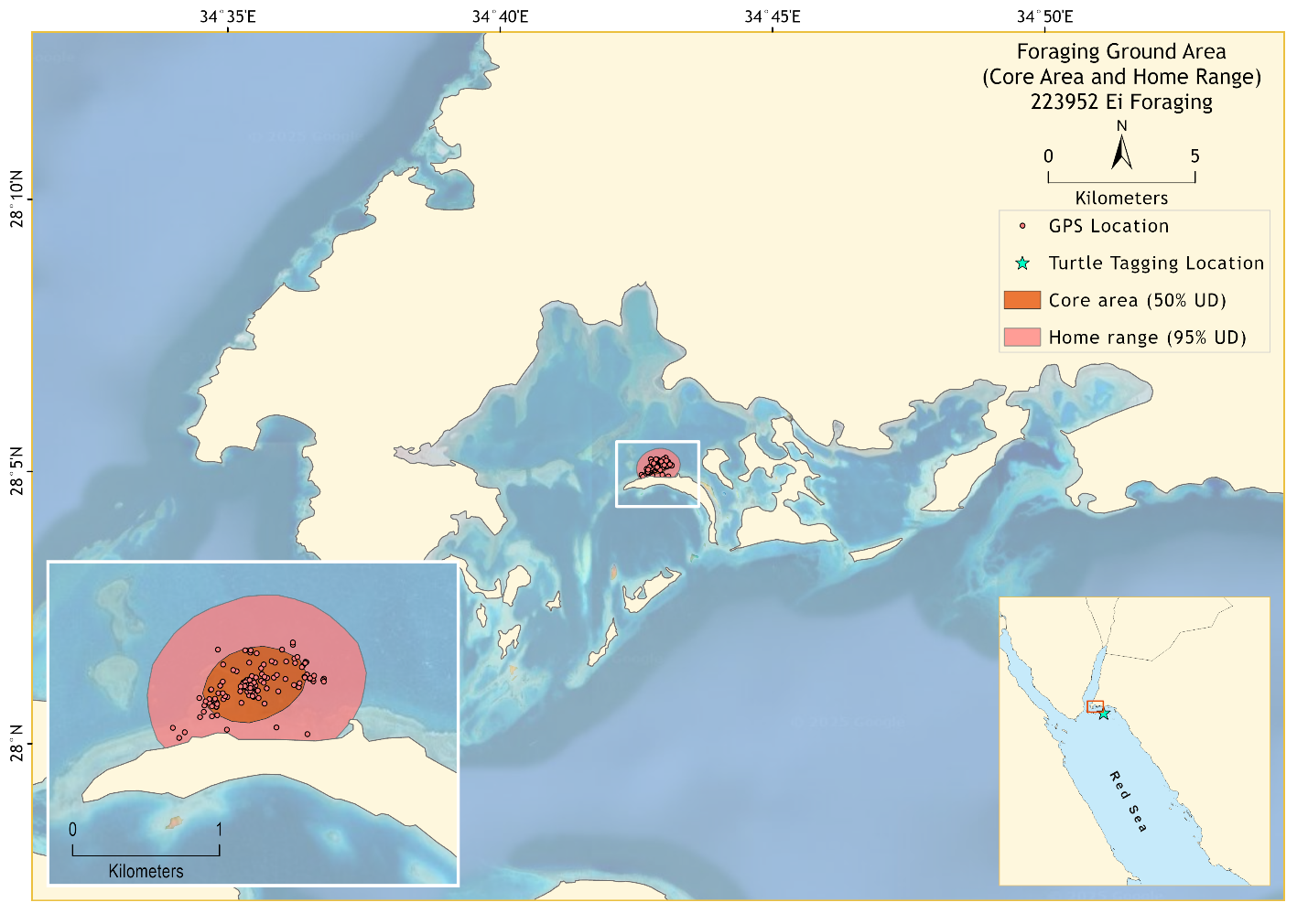


**Figure S17. PTT-0223953 Turtle Foraging Habitat Use: Core Area and Home Range (Utilization Distribution – UDs)**

***Eretmochelys imbricata***


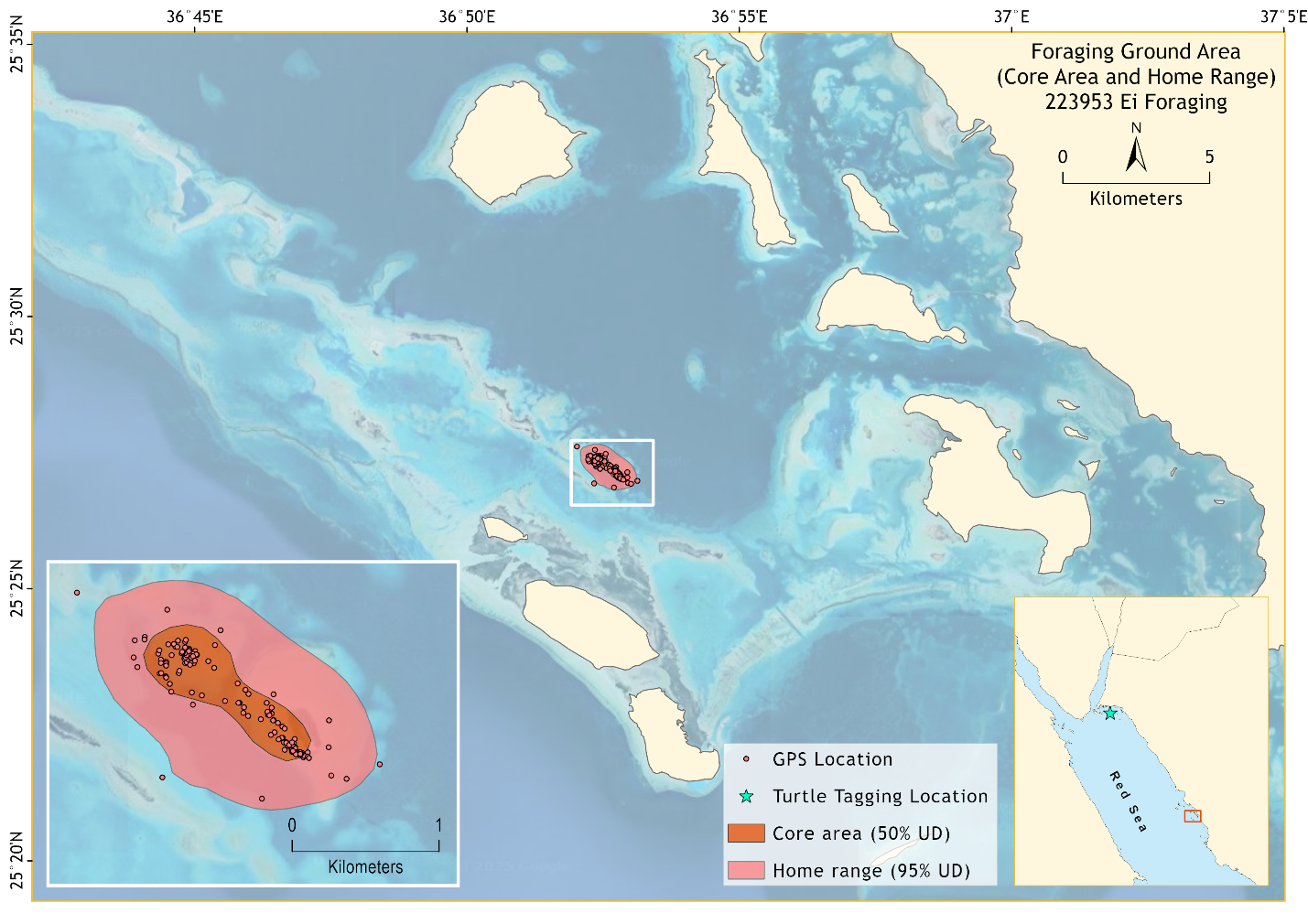


**Figure S18. PTT-0223955 Turtle Foraging Habitat Use: Core Area and Home Range (Utilization Distribution – UDs)**

***Eretmochelys imbricata***


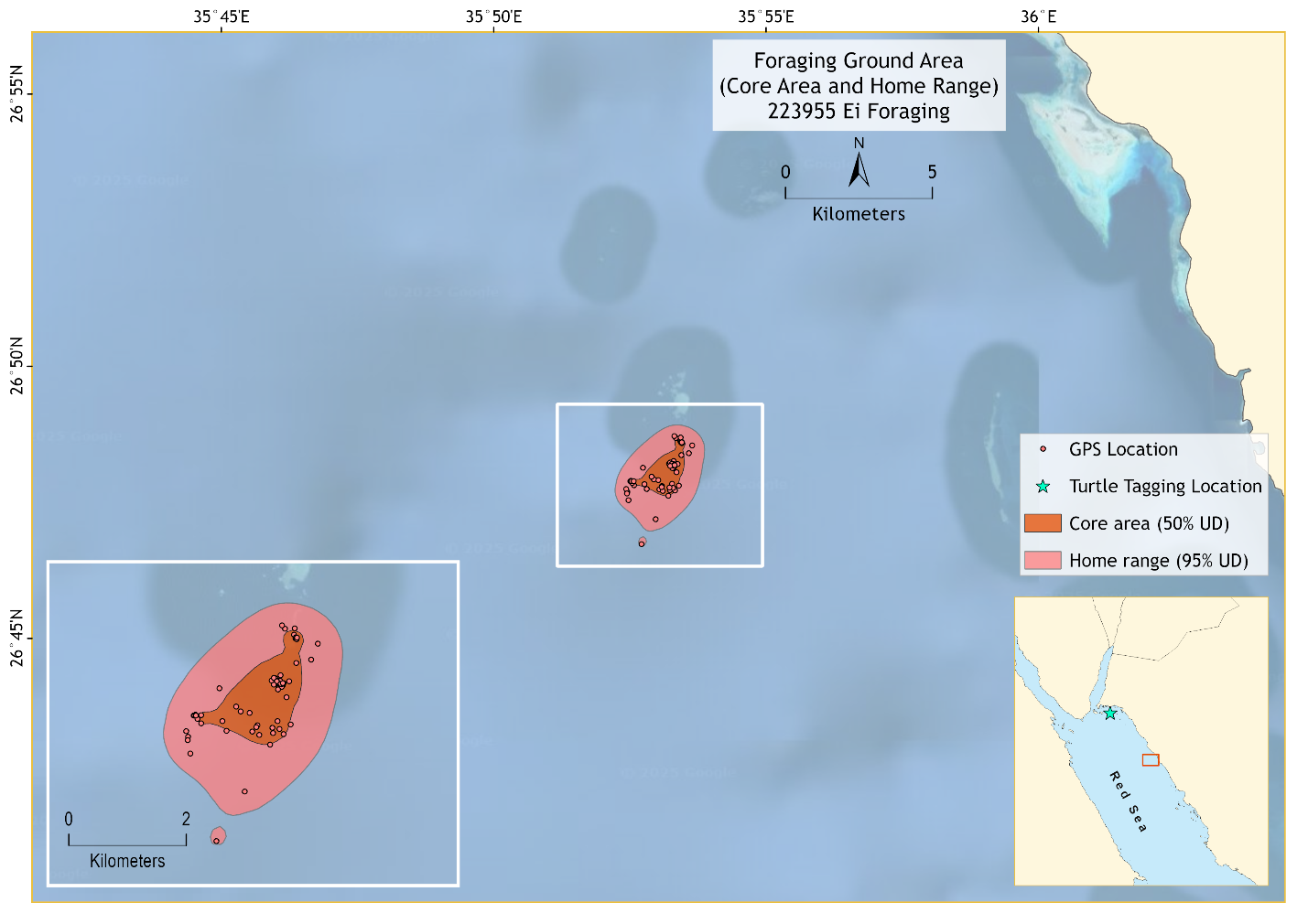


**Table S2. Duty Cycle Configuration of Lotek FastGPS Argos Tags Used in This Study**

| **Phase** | **Start Day** | **End Day** | **GPS Fix Interval (min)** | **Argos Uplink Limit (messages/day)** | **Retry Settings** |
| --- | --- | --- | --- | --- | --- |
| 0 | 0 | 60 | 15 | 400 | Up to 3 retries; 1-hour retry interval |
| 1 | 61 | End of battery life | 30 | 400 | Up to 3 retries; 1-hour retry interval |
